# Network pharmacology combined with molecular docking and experimental verification to elucidate the effect of flavan-3-ols and aromatic resin on anxiety

**DOI:** 10.1038/s41598-024-58877-z

**Published:** 2024-04-29

**Authors:** Ansari Vikhar Danish Ahmad, Subur W. Khan, Syed Ayaz Ali, Qazi Yasar

**Affiliations:** Y. B. Chavan College of Pharmacy, Dr. Rafiq Zakaria Campus, Aurangabad, Maharashtra India

**Keywords:** Computational biology and bioinformatics, Neurology

## Abstract

This study investigated the potential anxiolytic properties of flavan-3-ols and aromatic resins through a combined computational and experimental approach. Network pharmacology techniques were utilized to identify potential anxiolytic targets and compounds by analyzing protein–protein interactions and KEGG pathway data. Molecular docking and simulation studies were conducted to evaluate the binding interactions and stability of the identified targets. Behavioral tests, including the elevated plus maze test, open field test, light–dark test, actophotometer, and holeboard test, were used to assess anxiolytic activity. The compound-target network analysis revealed complex interactions involving 306 nodes and 526 edges, with significant interactions observed and an average node degree of 1.94. KEGG pathway analysis highlighted pathways such as neuroactive ligand-receptor interactions, dopaminergic synapses, and serotonergic synapses as being involved in anxiety modulation. Docking studies on EGCG (Epigallocatechin gallate) showed binding energies of −9.5 kcal/mol for MAOA, −9.2 kcal/mol for SLC6A4, and −7.4 kcal/mol for COMT. Molecular dynamic simulations indicated minimal fluctuations, suggesting the formation of stable complexes between small molecules and proteins. Behavioral tests demonstrated a significant reduction in anxiety-like behavior, as evidenced by an increased number of entries into and time spent in the open arm of the elevated plus maze test, light–dark test, open field center activity, hole board head dips, and actophotometer beam interruptions (p < 0.05 or p < 0.01). This research provides a comprehensive understanding of the multi-component, multi-target, and multi-pathway intervention mechanisms of flavan-3-ols and aromatic resins in anxiety treatment. Integrated network and behavioral analyses collectively support the anxiolytic potential of these compounds and offer valuable insights for future research in this area.

## Introduction

Anxiety disorders are complex mental health conditions characterized by recurrent and sudden episodes of unexplainable panic, fear, tension, and/or anxiety. These episodes often manifest alongside noticeable physiological symptoms such as palpitations, sweating, and disturbances in the autonomic nervous system. Its global incidence ranges from 3.8 to 25%, with approximately 70% of reported cases being chronic^1^. As one of the most prevalent mental health issues, anxiety disorders significantly impact individuals’ quality of life and societal balance^2^. In Western medicine, common treatment approaches include selective serotonin reuptake inhibitors (SSRIs), benzodiazepines such as diazepam, and other pharmacological agents^3,4^.

Benzodiazepines function by interacting with γ-aminobutyric acid (GABA) receptors. This interaction enhances GABAergic activity, leading to increased permeability of chloride ion channels. Consequently, there is a substantial influx of chloride ions into cells. This mechanism promotes neuronal cell hyperpolarization, inducing a central inhibitory effect crucial for alleviating symptoms of anxiety^5^. In contrast, selective serotonin reuptake inhibitors (SSRIs) operate by inhibiting presynaptic 5-HT reuptake. This action increases the concentration of serotonin (5-HT) in the synaptic cleft, facilitating enhanced transmission of 5-HT neurons and ultimately producing anxiolytic effects^6^. However, prolonged use of these medications is frequently linked to the development of drug dependence, cognitive impairment, and increased susceptibility to motor dysfunction^7^.

Exacerbated by delayed therapeutic effects, substantial rates of nonresponse, and the emergence of adverse effects such as nausea and headache, patients face significant challenges linked to the administration of the mentioned pharmacotherapies^8–10^. Thus, there is a need for the development of antianxiety medications with enhanced tolerability profiles and a decreased likelihood of adverse effects. Current treatments for anxiety disorders, like therapy and medication, show effectiveness but also have drawbacks such as partial effectiveness, side effects, and potential dependency. Moreover, not all patients respond well to these treatments, highlighting the need for personalized approaches. Existing treatments often focus on symptom management rather than addressing underlying mechanisms. Therefore, there's a need for new therapies that are more effective, tolerable, and tailored to the diverse nature of anxiety disorders, aiming for better outcomes. The worldwide emergence of the COVID-19 pandemic, first identified in December 2019, has been linked to a significant increase in psychological issues, such as heightened anxiety and despondency. This has led to notable public concern regarding mental health^11^. Subsequent investigations have revealed that sleep disturbances, anxiety, and depressive symptoms persist in individuals even six months after hospital discharge and subsequent recovery^12^. Traditional antianxiety medications often target specific molecules, requiring prolonged administration and increasing susceptibility to a range of side effects and potential dependency issues^13^. Given these challenges, there has been growing interest in exploring alternative medicinal approaches to address anxiety. The aim is to mitigate the adverse effects and unfavorable reactions associated with conventional Western medicine treatments. Despite the variety of anxiolytic agents available on the market, their effectiveness is limited by various inherent constraints and drawbacks^14,15^. Network pharmacology is a robust framework in today's biomedical landscape that effectively integrates and coordinates intricate networks involving drugs, targets, and diseases. This process facilitates a comprehensive understanding of complex pharmacological interactions^16–18^. This approach places significant importance on high-throughput screening, advanced network visualization, and thorough analysis, making it a crucial tool in advancing research in traditional medicine. In contemporary drug discovery, molecular docking, a widely used computational technique, plays a key role in elucidating drug functionality and mechanism. The tool accurately predicts the binding modality and corresponding binding free energy between target proteins and investigated compounds^19^. Significant progress has been made in investigating therapeutic interventions for various central nervous system disorders, such as Alzheimer's disease, anxiety, and depression as pregabalin was identified as a potential anxiolytic through molecular docking and pharmacophore modeling studies^20^. This underscores the crucial role of these studies in modern neuropsychopharmacological research^17,21,22^. The use of virtual screening, which heavily relies on molecular docking methodologies, has become indispensable in contemporary drug development. This approach facilitates the efficient and strategic identification of potential lead compounds^23^. Additionally, molecular dynamics (MD), a computational simulation method that integrates principles from physics, mathematics, and chemistry, has proven to be a powerful tool for in-depth exploration of protein dynamics. This can be achieved by tracking intricate changes in protein conformation over time^24,25^. This integrated approach provides a comprehensive understanding of the potential mechanisms underlying their effects on anxiety. Network pharmacology analyzes interactions between bioactive compounds and pathways related to anxiety, while molecular docking predicts their binding affinity to target proteins. Experimental validation confirms these predictions, enhancing the reliability of the findings. This multi-faceted approach not only highlights the therapeutic potential of flavan-3-ols and aromatic resin for anxiety but also advances our understanding of the molecular mechanisms involved, contributing to the development of novel anxiolytic agents with improved efficacy and safety profiles.

In this study, we aim to explore the potential effects of flavan-3-ols and aromatic resin in anxiety using in silico and in vivo techniques. The active compound targets were predicted and a drug-target interaction network was constructed. Additionally, molecular docking and dynamic simulations were used to validate the predicted targets and assess the binding affinity and stability of compound-target interactions, and in vivo, anxiety models were used to know the potential anti-anxiety effects. Understanding the molecular mechanisms of flavan-3-ols and aromatic resin in anxiety can help in the development of new treatments and target mechanisms for the prevention and treatment of this severe disease.

## Materials and methods

### Materials and reagents

Flavan-3-ols, including catechin and epigallocatechin gallate, were procured from Yucca Enterprises, which is located in Mumbai, Maharashtra, India. An aromatic resin, specifically Oudh, was obtained from Shabbar Dawasaz, located in Aurangabad, Maharashtra, India. The reference standard drug clonazepam was acquired from Abbott, India.

### Protein–protein interaction (PPI) network

Analysis of protein–protein interactions (PPIs) is a crucial tool for understanding the complex involvement of proteins in various biochemical cascades. This approach aids in obtaining a comprehensive understanding of cellular architecture, biological processes, and functional modalities. The investigation involved the use of the advanced virtual screening platform STRING 11.0 (https://string-db.org/)^26^, to explore the intricate network of interconnected proteins. The relevant genetic components were carefully uploaded to the STRING database, providing essential insights into multifaceted PPIs. The construction of the PPI network was meticulously tailored to the context of '*Homo* sapiens', and criteria for assessing the confidence level in interactions among target proteins were rigorously calibrated for the highest reliability, exceeding the threshold of data confidence set at 0.9. In this intricate network visualization, individual nodes represent distinct proteins, while the connecting edges intricately illustrate associations among diverse protein entities.

### Compound and disease-target genes

The initial crucial step in constructing the compound-target network involves identifying genes linked to the disease. Relevant data about anxiety-associated target genes were methodically collected from credible sources, including GeneCards^27^ (https://www.genecards.org) and the Disgenet Database (https://www.disgenet.org/search). Moreover, comprehensive information on the protein targets of flavan-3-ols and aromatic resin was obtained from SwissTargetPrediction (http://www.swisstargetprediction.ch/) and the Stitch database (http://stitch.embl.de/).

### Construction of a compound-target network

After conducting the protein–protein interaction analysis, the next step was to clarify the complex molecular mechanisms involved. This was achieved by building a detailed compound‒target network using Cytoscape^28^, visualization software version 3.7.1. This network structure is essential for understanding and analyzing the interactions between bioactive components and their specific targets. This approach helps to reveal the pathways involved in this complex biological network.

### GO and KEGG pathway annotation

Gene Ontology (GO) and Kyoto Encyclopedia of Genes and Genomes (KEGG) pathway annotation were carried out using the ShinyGo platform^29^. The GO analysis aimed to examine the gene cluster within the network, enhancing the precision of the data prediction. GO provides a systematically organized collection of standardized terms related to biological processes, molecular functions, and cellular components. It incorporates curated and predicted gene annotations derived from these terms across various species. The annotation of biological processes through GO serves as a resource for pathway enrichment analysis, facilitating the identification of essential biological processes within the context of the study objectives. Additionally, KEGG was utilized to explore the functions and metabolic pathways of the genes and molecules in the network under investigation. This study helps in elucidating contributory pathways associated with disease phenotypes, providing insights into the intricate interplay between molecular entities and disease mechanisms.

### Molecular docking

#### Software tools

The molecular docking analyses were performed using a diverse set of software and tools, such as PyRx-Virtual Screening Tool, Discovery Studio Visualizer 2020, AutoDock Vina, PyMOL, MGL Tools, the Protein Data Bank (PDB), and PubChem. The MD simulation was performed by using Desmond (Schrodinger LLC) on the 64-bit operating system, Processor: Core i7 12gen RAM 32 GB Graphics card NVIDIA RTX 4090.

#### Ligand preparation

The ligands and approved drug structure (clonazepam) were obtained from the U.S. National Library of Medicine PubChem official website (https://pubchem.ncbi.nlm.nih.gov/). Subsequently, the structures were imported into PyRx 0.8 through the open Babel tool, and energy minimization (optimization) was executed, taking into account essential parameters such as element, hybridization, and connectivity. This process utilized the universal force field^30^. The ligands were subsequently transformed into AutoDock Ligand format (PDBQT).

#### Target preparation

This study focused on the three-dimensional (3D) structures of SLC6A4 (PDB: 7LIA), COMT (PDB: 4XUC), and MAO-A (PDB: 2Z5X). The crystal structures were obtained from the RCSB Protein Data Bank (7LIA: https://www.rcsb.org/structure/7LIA; 4XUC: https://www.rcsb.org/structure/4XUC; 2Z5X: https://www.rcsb.org/structure/2Z5X). PyMol software (The PyMOL Molecular Graphics System, Version 2.4.1 Schrödinger, LLC) was used for the visualization of the downloaded structures. The target structures were optimized, purified, and prepared for docking using Discovery Studio Visualizer 2020. This involved the removal of unwanted water molecules and bound ligands from the protein structures, which were then saved in PDB file format in the same folder. To perform docking studies of the selected ligands and the approved drug (clonazepam), AutoDock Vina 1.1.2 in PyRx 0.8 was utilized^31^.

#### Docking procedure

The purified structures of the targets were imported into the docking software PyRx 0.8 using the "load molecule" option in the File toolbar. Subsequently, the receptor structure was transformed into an AutoDock macromolecule (PDBQT format) through the right-click option. Binding affinity studies were also conducted using the Vina Wizard Tool in PyRx 0.8. Both ligands and targets, in the form of PDBQT files, were chosen for the docking process. For the molecular docking simulation, specific three-dimensional grid boxes were established (size_x = 128.0322 Å, size_y = 133.9732 Å, and size_z = 133.7811 Å) for SLC6A4 (PDB: 7LIA); (size_x = −4.738983 Å, size_y = 3.468900 Å, and size_z = −22.898050 Å) for COMT (PDB: 4XUC); and (size_x = 37.958235 Å, size_y = 29.222957 Å, and size_z = −17.581270 Å) for MAO-A (PDB: 2Z5X). The AutoDock tool 1.5.6 was used for this purpose, with an exhaustiveness value set to 8. Following the selection of molecules, active amino acid residues were designated to outline the cavity utilizing the "Toggle Selection Spheres" option in PyRx. The grid box was appropriately aligned to encompass all active binding sites and essential residues. Subsequently, the ligands and targets were subjected to docking to determine their binding affinity.

### Identification of cavity and active amino acid residues

The protein's active amino acid residues were determined through the use of the BIOVIA Discovery Studio Visualizer (version 19.1.0.18287). The investigation of docking poses, as well as ligand and protein interactions, was conducted by importing the output files into PyMol software. This approach facilitated the identification of various types of interactions.

### Molecular dynamic simulation

Desmond, a software package developed by Schrodinger LLC, was utilized to conduct molecular dynamics (MD) simulations spanning 100 ns (ns). In the field of molecular dynamics simulation, the receptor-ligand docking methodology was employed to calculate rigid binding analyses for the chosen compounds for the target protein. MD simulation analyses were performed to predict the ligand binding status within a physiological environment by incorporating Newton's classical equation of motion. The chosen proteins and ligands underwent optimization and minimization procedures facilitated by Maestro's Protein Preparation Wizard, which involved addressing steric clashes, undesirable contacts, and distorted geometries. The System Builder tool was used to construct the systems, and TIP3P (Intermolecular Interaction Potential 3 Points Transferable), which features an orthorhombic box, served as the solvent model with the OPLS_2005 force field. Counter ions were introduced to neutralize the models, and 0.15 M sodium chloride was added to replicate physiological conditions. All of the systems were further equilibrated at a constant temperature of 300 K and a pressure of 1 bar by deploying NVT using the V-rescale thermostat and NPT using the Parrinello–Rahman barostat ensemble process for 100 ps at a constant temperature of 300 K and a pressure of 1 bar. The trajectories were stored at intervals of 100 picoseconds (ps) for subsequent analysis, and the stability of the protein–ligand complex was verified through root mean square deviation (RMSD) analysis over time^32^.

### Prime MM-GBSA analysis

The molecular mechanics generalized Born surface area (MM-GBSA) module of the prime was used to determine the binding free energy (Gbind) of the docked complex during MD simulations of SLC6A4 complexed with EGCG. Using the OPLS 2005 force field, VSGB solvent model, and rotamer search techniques, the binding free energy was estimated. The MD trajectory frames were chosen at intervals of 10 ns after the MD run. The total free energy binding was calculated using the Equation:

$${\text{dGbind }} = {\text{ Gcomplex }}{-} \, \left( {{\text{Gprotein }} + {\text{ Gligand}}} \right)$$

where, dGbind is the binding free energy, Gcomplex is the free energy of the complex, Gprotein is the free energy of the target protein, and Gligand is the free energy of the ligand.

### Preparation of flavans-3-ols and aromatic resin combinations

One hundred grams of aromatic resin (Oudh) was macerated with 30% ethanol at room temperature for 12 h. After maceration, the resulting extract underwent thorough filtration (Whatman International, Maidstone, UK). The filtered macerated extract was then evaporated at room temperature. The final formulation of the blend of flavan-3-ols and aromatic resin was prepared following the above process.

## Experimental validation

### Animals

All procedures involving animal experiments were reviewed and approved (IAEC/844/ac/04/CPCSEA) by the Institutional Animal Ethical Committee (IAEC) of Y. B. Chavan College of Pharmacy, Aurangabad, India. All the experiments were performed by the regulations and guidelines issued by the Committee for the Purpose of Control and Supervision of Experiments on Animals (CPCSEA) India. All experiments were performed according to relevant guidelines and regulations. Besides, the study is reported based on the ARRIVE guidelines. Swiss albino mice of either sex (M/F) with an initial weight within the range of 20 to 30 g were procured from the Wockhardt Research Centre Pvt. Ltd., situated in Aurangabad, Maharashtra, India. The animals were subsequently housed under standard environmental conditions characterized by a 12:12-h light–dark cycle, a controlled temperature of 25 ± 2 °C, and a relative humidity level of 55 ± 5%. These mice were provided ad libitum access to both standard food pellets and water. After a requisite acclimatization period of seven days, the mice were randomly partitioned into six groups, each comprising five individuals, as outlined in the Experimental Protocol (Table S1, Supplementary material).

### Acute toxicity study

In line with the guidelines set forth by the Organization for Economic Cooperation and Development (OECD), specifically OECD guideline No. 423, a randomized allocation was employed for the animal cohort, comprising two groups, each consisting of three mice. Group 1 served as the control group and was administered a daily oral dose of 2 ml of water. Group 2, identified as the treatment group, received a test dose of 2000 mg/kg flavan-3-ols and aromatic resin. This test dose adhered to the recommended limit test dose, as per the up-and-down procedure for determining the LD50. The extract was sequentially administered individually over 48 h. Each mouse in the treatment group was orally administered 2000 mg/kg of the extract, and water was used as the vehicle. The survival outcome of the first animal guided the administration of the same dose to the subsequent two animals. A vigilant monitoring regimen was implemented involving hourly surveillance for changes in behavior over 4 h and daily observations spanning fourteen days following OECD guidelines^33^.

### Elevated plus maze test

The elevated plus-maze (EPM) paradigm, commonly used in rodent models, functions as a dependable tool for detecting anxiety-like behavior. Its applicability extends to evaluating the anxiolytic potential of pharmaceutical agents, aiding in the understanding of the underlying mechanisms related to anxiety regulation^34^. Following the established protocol, each mouse underwent a 5-min exposure to the maze. Subsequently, the number of entries into both the open and closed arms, along with the duration of time spent in each arm, were systematically documented utilizing Any-maze v7.2 video tracking software (Stoelting Co., USA). This meticulous approach ensured precision and accuracy in the acquisition of data^35^.

### Light and dark test

The light and dark test was performed by the established protocol outlined by^36^, with minor modifications. Thirty minutes after the elevated plus-maze (EPM) test, the experimental subjects were evaluated in a specially designed apparatus consisting of distinct bright and dark compartments. The dimensions of the apparatus were set at a ratio of 1:3 for the dark section and 2:3 for the light section, measuring 46 cm (length) × 27 cm (width) × 30 cm (height) overall. During the test, the mice were centrally positioned within the light compartment, with their dorsum facing the dark compartment. The number of entries made by the mice into the light and dark compartments, as well as the duration of time spent in each compartment, were carefully recorded over 5 min using Any-maze v7.2 video tracking software (Stoelting Co., USA). Following the test, the mice were gently removed from the apparatus by grasping their tails at the base and were then returned to their respective home cages. To maintain consistency, the maze apparatus was thoroughly cleaned between each experimental trial using a 10% ethanol solution and allowed to air dry, following the methodology described by^37^.

### Open field test

Each mouse was housed in a standardized acrylic enclosure measuring 50 × 50 × 10 cm^38^. The open field apparatus was designed with 25 squares, consisting of nine internal squares positioned centrally and 16 squares located along the periphery near the enclosure walls. After an oral administration period lasting one hour involving the application of the specified vehicle, clonazepam, flavan-3-ols, or aromatic resin treatment combination, the mice were individually placed in one of the predetermined corner squares. Observations were carried out for a duration of five minutes, during which Any-maze v7.2 video tracking software (Stoelting Co., USA) was used to document and assess various parameters. These parameters included the number of entries into the central zone, the total duration spent within the central zone, the number of entries made within the peripheral zone, and the cumulative time spent within the peripheral zone.

### Hole board test

The setup used in the experiment consisted of a wooden enclosure measuring 40 × 40 × 25 cm. The apparatus included a raised platform designed to hold 16 evenly spaced holes, each with a diameter of 3 cm. To maintain consistent conditions, the apparatus was elevated 25 cm above the ground. Test mice were given oral doses of flavan-3-ols and aromatic resin at 50 mg/kg. These doses were administered an hour before the mice were placed in the described behavioral setup. The assessment of exploratory behavior involved recording the frequency of head pokes over a specific 5-min observation period. Notably, the comparison included the use of a standard anxiolytic agent, clonazepam, given orally at a dose of 1 mg/kg, which served as a reference point^39^.

### Locomotor activity

In our research, we utilized an actophotometer to accurately monitor the locomotor activity of the experimental subjects. The actophotometer records and digitally displays interruptions in a light beam caused by the subjects' movements, with each interruption representing a specific activity event. To establish baseline activity levels, each mouse underwent a five-minute session with the actophotometer to determine its initial activity score. Subsequently, the mice were orally gavaged with a combination of flavan-3-ols and aromatic resin at a dose of 50 mg/kg. Additionally, a standard pharmaceutical, clonazepam, was orally administered at a dosage of 1 mg/kg as a reference therapy. After a 1-h interval, the mice were once again placed in the actophotometer to evaluate their post-treatment activity scores. This methodology is in accordance with the approach outlined by^40^.

### Statistical analysis

Statistical analysis was conducted employing Graph Pad Prism 9.5.1 software (Graph Pad Software, San Diego, CA, USA). The data are presented as the means ± standard errors (SEs). Group comparisons were assessed through analysis of variance (ANOVA) utilizing a one-way test, followed by Dunnett's multiple comparison test. A *p*-value < 0.05* was deemed statistically significant, a p-value < 0.01** was considered highly significant, a p-value < 0.001*** was denoted as extremely significant, and a p-value < 0.001**** was denoted as exceptionally significant while 'ns' indicated non-significance.

### Ethical approval

All the experiments were performed as per the Institutional Animal Ethics Committee (IAEC) of Y.B. Chavan College of Pharmacy, Aurangabad (IAEC/844/ac/04/CPCSEA). All experiments were performed in accordance with relevant guidelines and regulations. Besides, the study is reported based on the ARRIVE guidelines.

## Results

### Acute toxicity study

The Coadministration of flavan-3-ols and aromatic resin did not induce any discernible alterations in behavior or lead to mortality even at a high dose of 2000 mg/kg, as indicated in Table S2 (Supplementary material).

### Construction and analysis of the target PPI network

**Figure 1:**
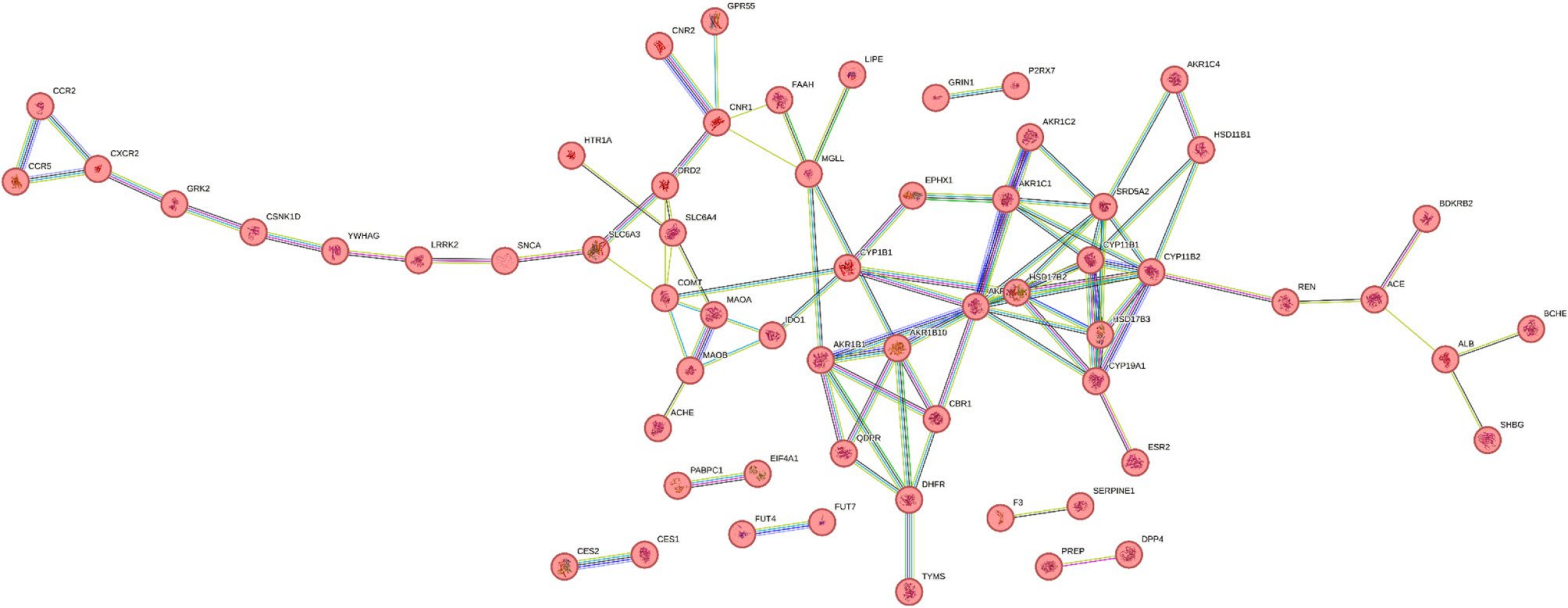
Protein–protein interaction network of flavan-3-ols and aromatic resin in anxiety targets obtained from STRING v_11.0 database.

The target genes associated with the respective components were thoroughly examined using STRING v_11 for the construction and visualization of the protein‒protein interaction (PPI) network. The high-confidence data on target protein interactions were filtered using a stringent threshold set at a score level exceeding 0.9. The resulting PPI network, as illustrated in Fig. 1, consisted of 98 nodes and 95 edges, where each edge represented distinct protein‒protein interactions (PPIs). Notably, the average node degree, which reflects the number of connected targets within the network, was determined to be 1.94. A local clustering coefficient of 0.388 corresponds to the number of targets connected to the network. The analysis of the PPI network highlighted the involvement of key targets, including MAOA, MAOB, COMT, DRD2, HTR1A, ACHE, GPR55, and SLC6A4, in the regulation of anxiety. Moreover, the spatial distributions of MAOA, MAOB, COMT, and SLC6A4 on the left side of the network suggested that these genes significantly contributed to the pathogenesis of anxiety.

### Arrangement and construction of disease-target network

**Figure 2:**
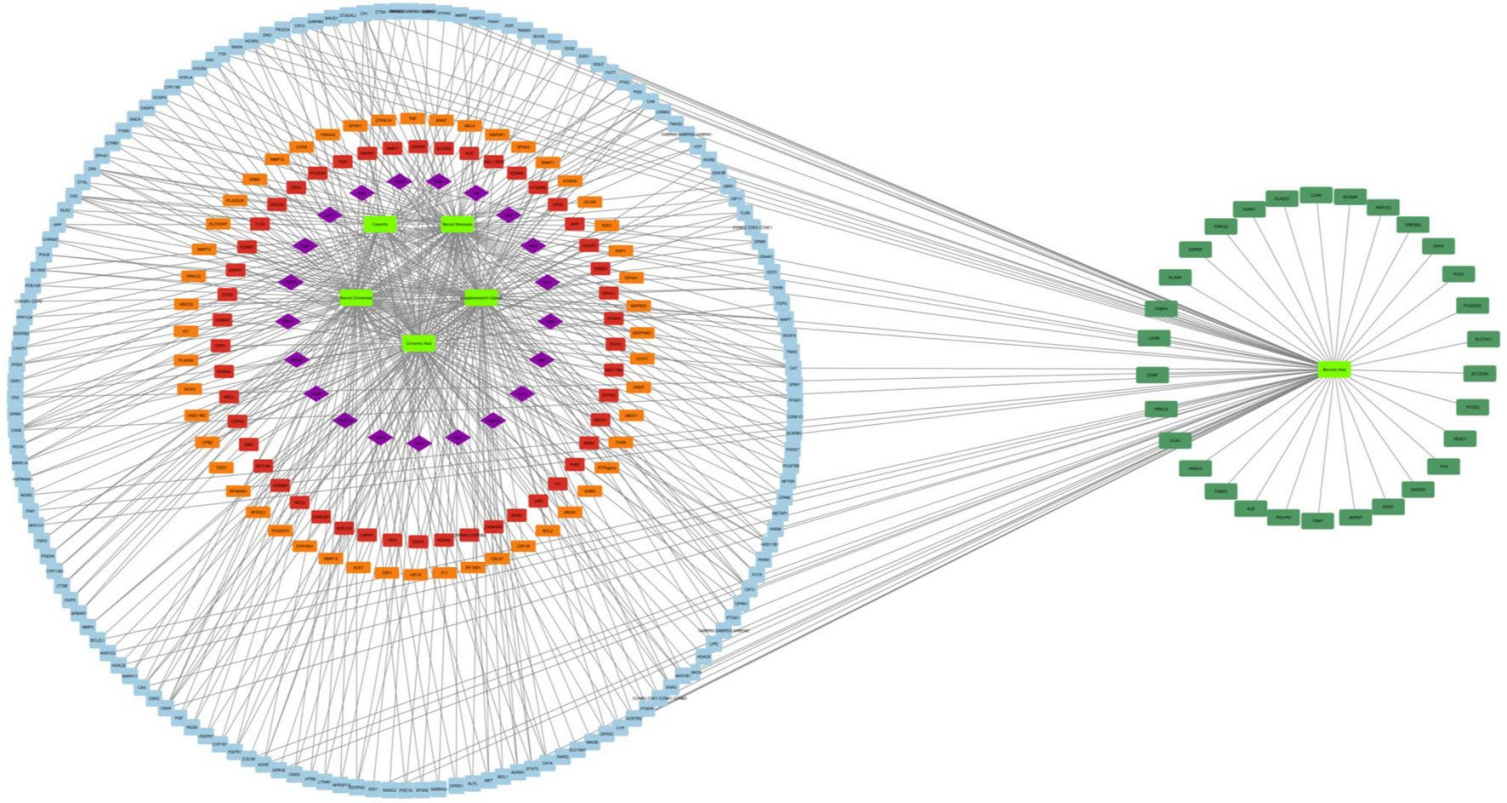
Compound-target-anxiety network constructed by Cytoscape v_3.7.1.

To explore the signaling pathways and functional implications of the chosen target genes, we utilized Cytoscape to import and analyze the data. This process facilitated the development of a detailed compound-target network. The network, depicted in Fig. 2, outlines the intricate mechanisms involved in the pharmacological effects of the compounds concerning anxiety treatment. This database included six distinct ingredients and showed case interactions with 526 target proteins. Notably, network analysis emphasized the convergence of multiple components on various targets, suggesting potential synergistic modulation by active biochemical entities. This complex interplay may contribute not only to the therapeutic efficacy of these agents in anxiety management but also to the treatment of other associated diseases and disorders. Detailed information about the network's topological parameters is available in Table S3 of the Supplementary material, emphasizing the crucial role of each target in the intricate network architecture. Understanding topological parameters is vital in network pharmacology, providing quantitative measures to characterize the structure, function, and dynamics of complex biological networks. Such information proves valuable in identifying potential drug targets, unraveling disease mechanisms, and guiding therapeutic interventions.

### GO enrichment analysis and KEGG pathway annotation

**Figure 3:**
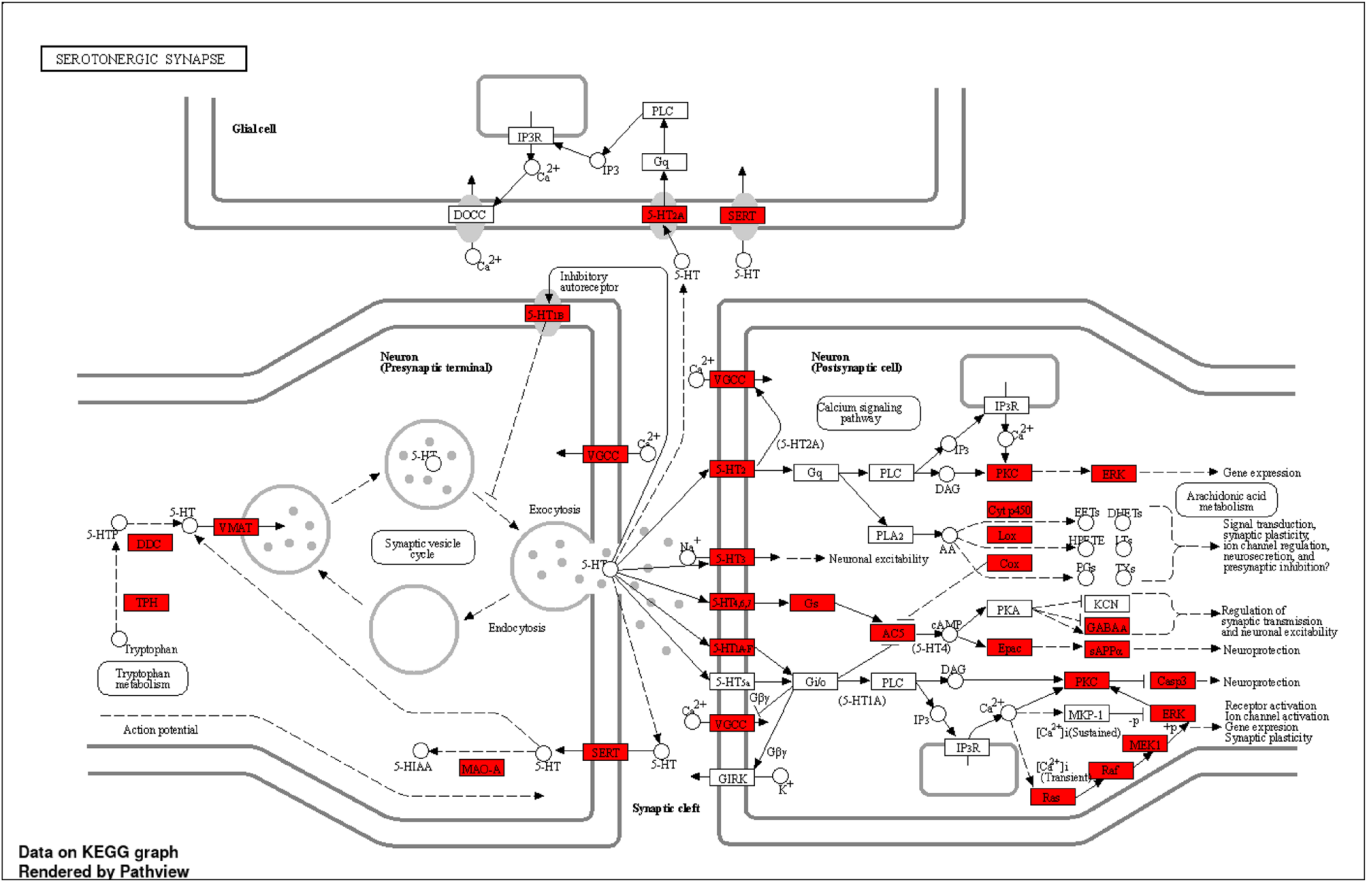
Serotonergic synapse.

The investigation of underlying target proteins involved the utilization of GO enrichment analysis. The ShinyGO settings were configured with three criteria, focusing on GO biological (Table 1 and Fig. S1A), GO molecular (Table 2 and Fig. S1B), and GO cellular (Table 3 and Fig. S1C) processes. Special attention was given to the crucial KEGG pathways (Table 4 and Fig. S1D). The fusion of GO terms was constrained to a threshold of p ≤ 0.05, following the false discovery rate determined via the Benjamini–Hochberg method. Comprehensive GO and KEGG analyses were also conducted to elucidate the signaling pathways involved, revealing significant associations with serotonergic synapses (Fig. 3), neuroactive ligand-receptor interactions, and dopaminergic synapses (Fig. S2 A-B). Flavan-3-ols and aromatic resin exhibit potential application in managing various conditions, such as nicotine addiction, amphetamine addiction, long-term depression, bladder cancer, morphine addiction, and Kaposi sarcoma-associated herpes virus infection. Originally chosen for their exploration of anxiety-related effects, thorough KEGG analysis (Fig. S1D) revealed the involvement of these genes in multiple disease pathways and disorders. Given the intricate interactions and the depicted network, the use of flavan-3-ols and aromatic resin as novel pharmacotherapeutic agents shows promise for treating diverse diseases and disorders.

**Table 1:** Go Biological process.

Description	Count in gene set	False discovery rate
Behavior	164	1.09 × 10^–89^
Modulation of chemical synaptic transmission	113	1.45 × 10^–55^
Reg. of trans-synaptic signaling	113	1.76 × 10^–55^
Chemical synaptic transmission	163	3.08 × 10^–73^
Anterograde trans-synaptic signaling	163	3.08 × 10^–73^
Trans-synaptic signaling	164	2.90 × 10^–73^
Synaptic signaling	169	2.61 × 10^–74^
Cellular response to organonitrogen compound	137	5.22 × 10^–57^
Reg. of system proc	118	6.26 × 10^–48^
Cellular response to nitrogen compound	145	2.26 × 10^–57^

**Table 2:** Go molecular process.

Description	Count in gene set	False discovery rate
Carbonate dehydratase activity	12	2.28 × 10^–13^
Neuropeptide hormone activity	18	6.14 × 10^–17^
Neuropeptide receptor binding	17	1.04 × 10^–13^
Nuclear receptor activity	23	6.27 × 10^–16^
Ligand-activated transcription factor activity	23	6.27 × 10^–16^
Hormone binding	31	9.13 × 10^–20^
Neuropeptide receptor activity	16	5.14 × 10^–10^
Peptide hormone binding	17	2.66 × 10^–10^
Scaffold protein binding	19	4.19 × 10^–11^
Hormone activity	37	7.20 × 10^–20^
Postsynaptic neurotransmitter receptor activity	19	1.86 × 10^–10^

**Table 3:** Go cellular components.

Description	Count in gene set	False discovery rate
Integral component of presynaptic membrane	22	1.74 × 10^–13^
Axon terminus	39	7.12 × 10^–23^
Neuron projection terminus	44	1.32 × 10^–25^
Neuron projection membrane	19	3.35 × 10^–11^
Terminal bouton	17	5.98 × 10^–10^
GABA-ergic synapse	19	5.59 × 10^–10^
Presynaptic membrane	36	2.06 × 10^–17^
Perikaryon	38	1.28 × 10^–17^
Integral component of postsynaptic membrane	27	1.17 × 10^–12^
Integral component of synaptic membrane	34	1.60 × 10^–15^

**Table 4:** KEGG pathways.

Description	Count in gene set	False discovery rate
Bladder cancer	18	2.28 × 10^–14^
Nicotine addiction	17	2.20 × 10^–13^
EGFR tyrosine kinase inhibitor resistance	31	4.19 × 10^–22^
Prolactin signaling pathway	23	7.31 × 10^–15^
Prostate cancer	31	2.85 × 10^–19^
Amphetamine addiction	22	5.12 × 10^–14^
Neuroactive ligand-receptor interaction	111	1.62 × 10^–68^
Long-term depression	19	2.89 × 10^–12^
Dopaminergic synapse	28	7.58 × 10^–13^
Serotonergic synapse	33	2.85 × 10^–19^

### Molecular docking

**Figure 4:**
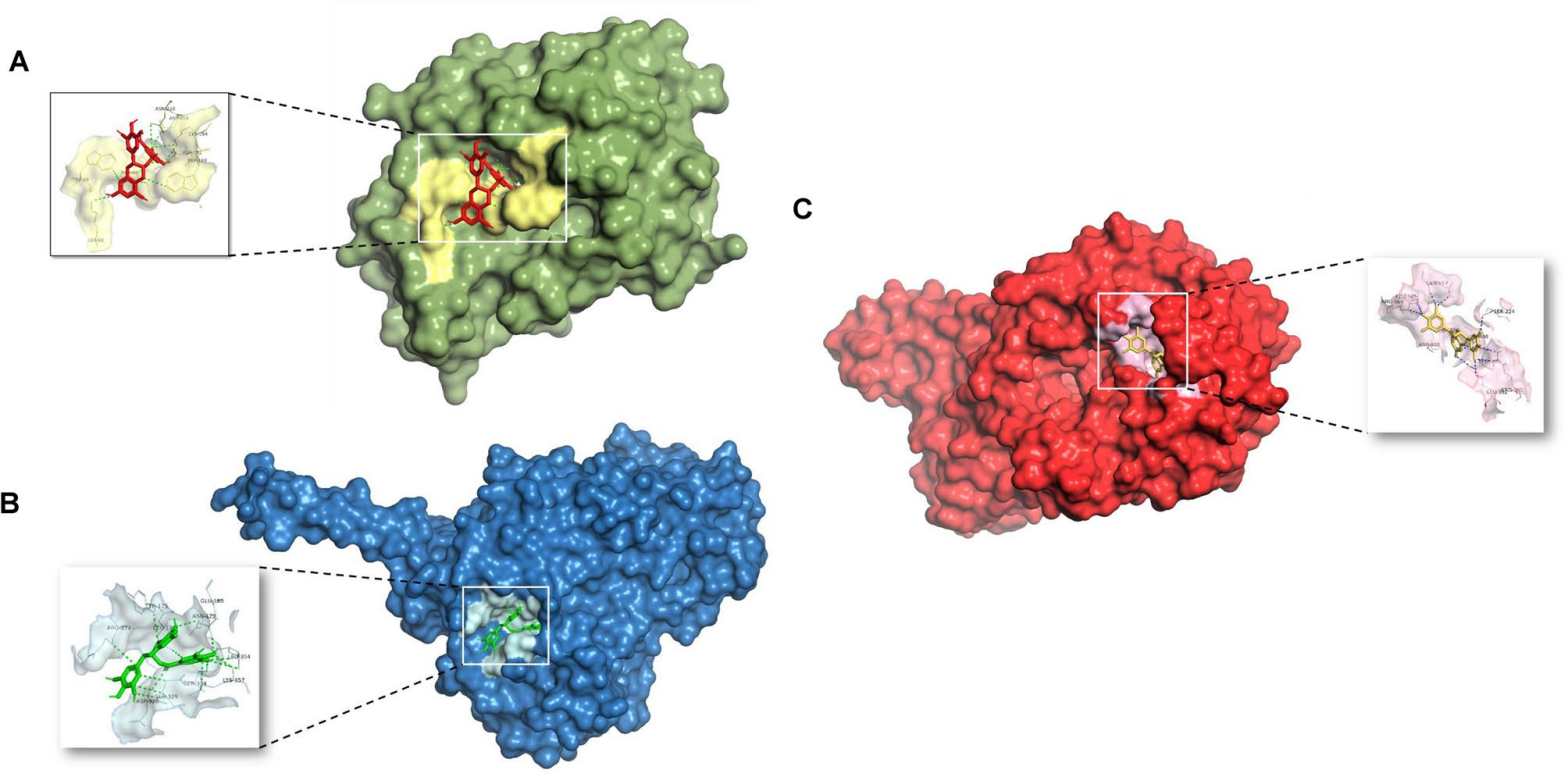
Amino acid interactions formed between docked complexes of (**A**) epigallocatechin gallate with COMT (PDB: 4XUC), (**B**) epigallocatechin gallate with MAO-A (PDB: 2Z5X), (**C**) EGCG with SLC6A4 (PDB: 7LIA).

Following a comprehensive examination of intricate signaling pathways and pathological conditions associated with specific genes, it is essential to explore the structure-based design of molecular components. This exploration includes an assessment of the ability of these ligands to predict the binding conformation of low-molecular-weight ligands to target binding sites. The rationale for selecting these proteins lies in their significant involvement in protein‒protein interactions (PPIs), compound–target network construction, and KEGG analysis. There is also a specific emphasis on the crucial roles of these proteins in the complex mechanism underlying anxiety. Detailed insights into the docking scores and conformations of the active components, including catechin, epigallocatechin gallate, benzyl benzoate, benzoic acid, benzyl cinnamate, and cinnamic acid, concerning their interactions with COMT (PDB: 4XUC), MAOA (PDB: 2Z5X), and SLC6A4 (PDB: 7LIA) are presented in Tables 5 and 6 and are visually represented in (Fig. 4A–C).

**Table 5:** Docking scores of flavan-3-ols and aromatic resin compounds with potential targets.

Compound	PubChem ID	Binding energy (kcal/mol)		
		COMT (PDB: 4XUC)	MAOA (PDB: 2Z5X)	SLC6A4 (PDB: 7LIA)
Epigallocatechin gallate	65,064	−**7.4**	**−9.5**	**−9.2**
Catechin	9064	−7.1	−8.5	−8.4
Benzyl benzoate	2345	−5.9	−8.7	−7.8
Benzyl cinnamate	5,273,469	−7.3	−7.8	−8.3
Benzoic acid	243	−5.8	−6	−6
cinnamic acid	444,539	−6	−6.2	−6.6
Clonazepam	2802	**−6.3**	**−7.2**	**−6.9**

Significant values are in bold.

**Table 6:** Estimated free energy of binding, H-bond interactions, hydrophobic interactions and other interactions between compounds and receptors.

Targets	Compounds	Interacting residues				
		H-bonding interactions		Hydrophobic interactions	π-stacking	Salt bridges
SLC6A4	Epigallocatechin gallate	SER224, ARG390, GLU392, ASP400, SER404, THR409, ARG564, LEU565, GLN567		ILE408	–	–
	Benzoic_acid	ALA96		TYR95, ASP98, ILE172	PHE341	–
	Benzyl_benzoate	THR497		ALA331, PHE335, PHE556, PRO561	PHE556	–
	Catechin	ARG104, THR497, SER559		ALA331, PHE335	PHE556	–
	Benzyl cinnamate	THR497		ILE108, ALA331, GLN332, PHE556	PHE556	ARG104
	Cinnamic_acid	TYR95, PHE335		ILE172, TYR176, PHE341	–	–
	Clonazepam	GLU494		TYR107, ILE108, ALA331, GLN332	–	–
COMT	Epigallocatechin	LYS55, ASP191, LYS194, ASN220		TRP88, MET90, TRP193	–	LYS194
	Benzoic_acid	GLY116, SER169		ILE141, HIS192	TRP193	
	Benzyl_benzoate	ARG135		VAL111, ARG135, ILE137, THR163, TYR180, VAL182	–	–
	Catechin	ASP191, LYS194, ASP195, ASN220		TRP193, LYS194	LYS194	
	Benzyl cinnamate	–		TRP88, ILE141, TRP193, LEU248	TRP193	LYS194
	Cinnamic_acid	GLU140		ILE141, HIS192		
	Clonazepam	–		TRP88, MET90, TRP	LYS194	–
MAOA	Epigallocatechin	ASN179, GLU327, ASP328, GLU329, SER334, LEU354, LYS357		ARG172, TYR175, LEU176, GLU329, LYS357	TYR175	LYS357
	Benzoic_acid	GLN215, TYR407	PHE352, TYR444		TYR407	–
	Benzyl_benzoate	TYR407		TYR69, ILE180, GLN215, LEU337, PHE352, TYR407	TYR407	–
	Catechin	ALA44		ALA44, PRO243, LEU277, LYS280, ILE281, TYR402	–	–
	Benzyl cinnamate	–		ILE19, ALA44, THR245, ILE273, LEU277, LYS280, TYR402	–	–
	Cinnamic_acid	ASN125, GLU492		PHE112, TYR121, TYR124, ARG129, GLU492	–	–
	Clonazepam	HIS488		PHE112	–	–

### Molecular dynamic simulation

**Figure 5:**
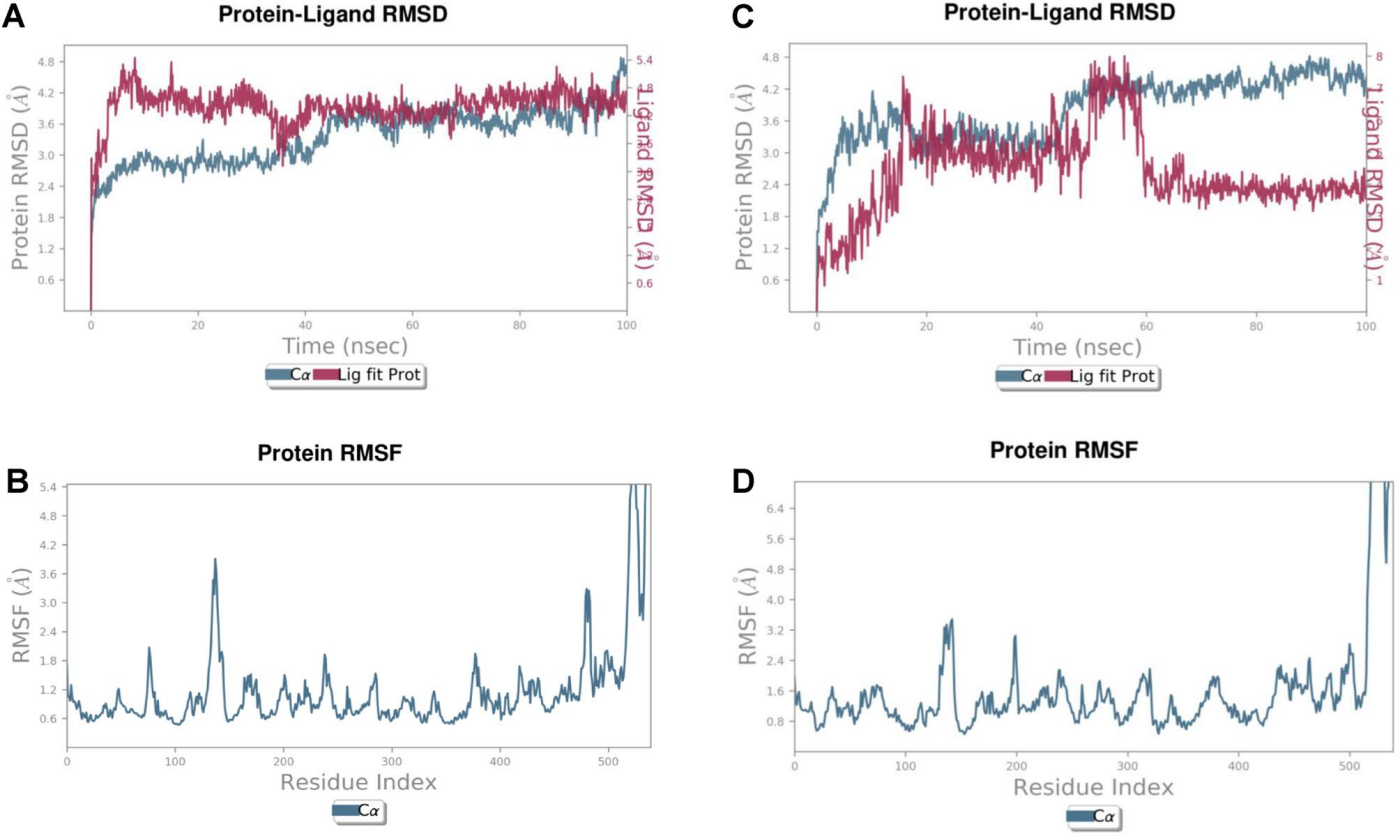
RMSD/RMSF values during molecular dynamics simulation (**A,B**) EGCG-7LIA complex, (**C,D**) Clonazepam-7LIA complex.

Considering the results of network pharmacology and molecular docking, SLC6A4 was selected for molecular dynamics simulation to further study the interaction between the active compound epigallocatechin gallate (EGCG) and the target protein. The estimation and comparison of the stability, conformational changes, and residual fluctuations in the complexes and empty protein over the 100 ns simulation were performed using a molecular dynamic simulation. The simulation data were analyzed for trajectories, and the resulting trajectories of the simulated complexes were inspected for different standard simulation parameters, such as RMSDs, root-mean-square fluctuations (RMSFs), intermolecular interactions, protein–ligand contacts, solvent-accessible surface area (SASA) and radius of gyration (rGyr). A 100 ns molecular dynamics simulation of the docked pose of the EGCG-7LIA complex revealed that the RMSD of the protein Cα atoms (Fig. 5A) stabilized after ligand binding, showing minute fluctuations. The ligand RMSD (Fig. 5A) initially exhibited partial fluctuations at 0–8 ns, with an RMSD of 1.8–4.8 Å, which then stabilized until 100 ns, showing an average RMSD of 3 to 4.2 Å. In comparison to the ligand, (Fig. 5C) shows RMSD curves for the clonazepam, which shows initial fluctuations up to 18 ns, with an RMSD of 0.6–4.4 Å, which then stabilized until 48 ns and further fluctuated to 58 ns and then stabilized until 100 ns. The RMSF (Fig. 5B) of the protein was stable except for amino acids initially from 130 to 150, which exhibited minor fluctuations until 4 Å, which were present in the loops and subsequently stabilized. Finally, the protein appeared stable except for amino acids 510 to 530, which exhibited greater fluctuations, whereas (Fig. 5D) shows an RMSF plot for the clonazepam similar to that of EGCG.

From the 2D ligand interaction diagram (Fig. S3A) of the 100 ns simulation, docked pose of EGCG- 7LIA complex revealed and exhibited strong hydrogen bonds with ASP400 of 95% and 92%, SER226 of 67%, GLU392 of 64% and LYS399 of 41% of total simulation trajectory as compared with the standard clonazepam which shows weak hydrogen bonding with GLU494 of 83% (Fig. S3B). Additionally, hydrophobic bonds were observed between TRP220, LEU222, ARG390, ALA398, LYS399, ILE408, and LEU565 on the stimulation trajectory, and extensive water bridge interactions were formed with the ligand protein contacts ASN211, TRP220, LEU222, SER224, ARG390, GLU392, GLU396, VAL397, LYS399 and ARG564. Whereas hydrophobic bonds in clonazepam were observed between TYR107, ILE108, GLN332, and extensive water bridge interactions were formed with the clonazepam protein contacts are TRP103, ARG104, TYR107, GLN111, ASP328, LYS399, ASP400, GLU402, GLU493, GLU494, THR497, SER555, SER559, GLN562, ARG564. The ligand interaction fraction (Fig. S4A) revealed that EGCG strongly interacted with ASN211, TRP220, THR225, SER226, GLU229, ARG390, GLU392, GLU396, ALA398, LYS399, ASP400, SER404 and GLN567 in the simulation process, suggesting that these hydrogen bonds are critical for stabilizing EGCG in the protein pocket. While the ligand interaction fraction of clonazepam (Fig. S4B) showed less interaction with GLN111, GLY402, GLU494, PHE556, and GLN562. Additionally, EGCG exhibited strong hydrophobic interactions with TRP220 and ILE408, which may also stabilize the complex, while standard clonazepam shows hydrophobic interactions with TYR107, ILE108, ALA331, ALA401, LYS419, and PHE556. The interaction time of each amino acid residue is given in (Fig. S5A). Notably, the interaction times of the amino acid residues TRP220, SER226, GLU229, ARG390, GLU392, LYS399, and ASP400 were greater than those of all the other amino acids. The interaction of amino acid THR225 was steady for 90 ns, after which the interaction was lost. The interaction of amino acid SER404 was steady for 10 ns, after which the interaction stopped. The interaction between THR221 and ALA404 occurred after 22 to 100 ns and fewer interactions were found. The interaction time of each amino acid residue for the clonazepam is shown in (Fig S5B). The amino acid GLU494 interacted initially for 7 ns and then there was loss of interaction up to 10 ns and then the interaction was steady up to 100 ns. Protein secondary structure elements (SSEs), such as alpha helices and beta strands, are monitored for both EGCG and clonazepam throughout the simulation. The plot shows the SSE distribution by residue index throughout the protein structure (Fig. S6) summarizes the SSE composition for each trajectory frame over the course of the simulation, and the plot at the bottom monitors each residue and its SSE assignment over time. During the simulation, the MolSA and SASA of the SLC6A4 and docked complexes of the EGCG and clonazepam were examined and plotted (Fig. S7A, B). SASA is another key attribute to investigate in MD simulation experiments to better understand biomolecular complex conformational stability. Throughout the 100 ns MD simulation period, SASA plots of both EGCG-7LIA and clonazepam-7LIA complexes were fairly equilibrated. The EGCG-7LIA complex was exposed to the area of 90–150 Å^2^. While the Clonazepam-7LIA complex was exposed to a surface area of 80–240 Å^2^. Molecular surface calculation with 1.4 Å probe radius. This value is equivalent to a van der Waals surface area. The average MolSa values for EGCG and Clonazepam were found to be 366–378 Å^2^ and 267–273 Å^2^ respectively. The overall ligand properties for both EGCG and clonazepam are shown in (Fig. S7A, B). The minimized binding free energies of the complexes were calculated using the MM-GBSA method. Table S4 shows the Supplementary material.

### In vivo experimental validation

#### Elevated plus maze

**Figure 6:**
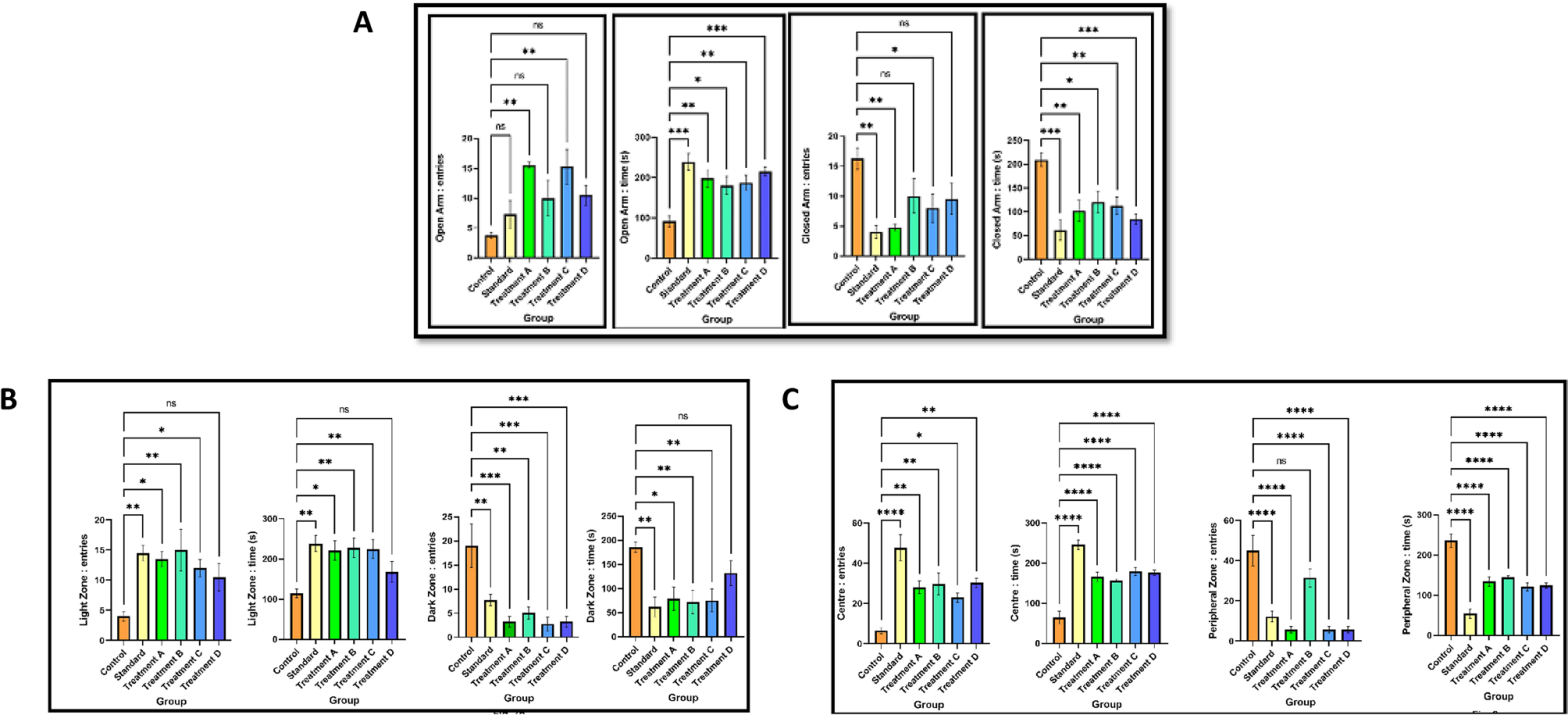
(**A**) Elevated plus maze (a) the open arm entries, (b) the open arm time, (c) the close arm entries, (d) the close arm time in a 5 min session of EPM was recorded. (**B**) Light and dark test (a) the light zone entries, (b) the light zone time, (c) the dark zone entries, (d) the dark zone time in a 5 min session pf D/L was recorded. (**C**) Open field test (a) the center entries, (**b**) the center time, (**c**) the peripheral zone entries, (**d**) the peripheral zone time in a 5 min session of OFT was recorded.

The results indicated a significant increase (p < 0.05) in the number of entries and the time spent in the open arms, while the number of entries and time spent in the closed arms significantly decreased (p < 0.05) at a dosage of 50 mg/kg and for clonazepam at an oral dose of 1 mg/kg (Fig. 6A).

#### Light and dark test

As shown in (Fig. 6B), the number of entries in the light zone and the time spent in the light zone significantly increased (p < 0.05), while the number of entries in the dark zone and the time spent in the dark zone notably decreased (p < 0.01) at an oral dose of 50 mg/kg. Additionally, the administration of clonazepam at an oral dose of 1 mg/kg resulted in a significant (p < 0.05) increase in the percentage of time spent in the light compartment and a significant (p < 0.05) decrease in the dark compartment.

#### Open field test

Administration of a 50 mg/kg p.o. dosage resulted in a significant (p < 0.001) increase in the number of entries and time spent in the center zone, while concurrently leading to a considerable (p < 0.001) decrease in the number of entries and time spent in the peripheral zone. Similar results were observed with the administration of clonazepam at a p.o. dosage of 1 mg/kg, which significantly (p < 0.001) increased the number of entries and percentage of time spent in the central zone, as well as a notable decrease in peripheral zone entrance and time spent in the peripheral zone (Fig. 6C).

#### Hole board test

**Figure 7:**
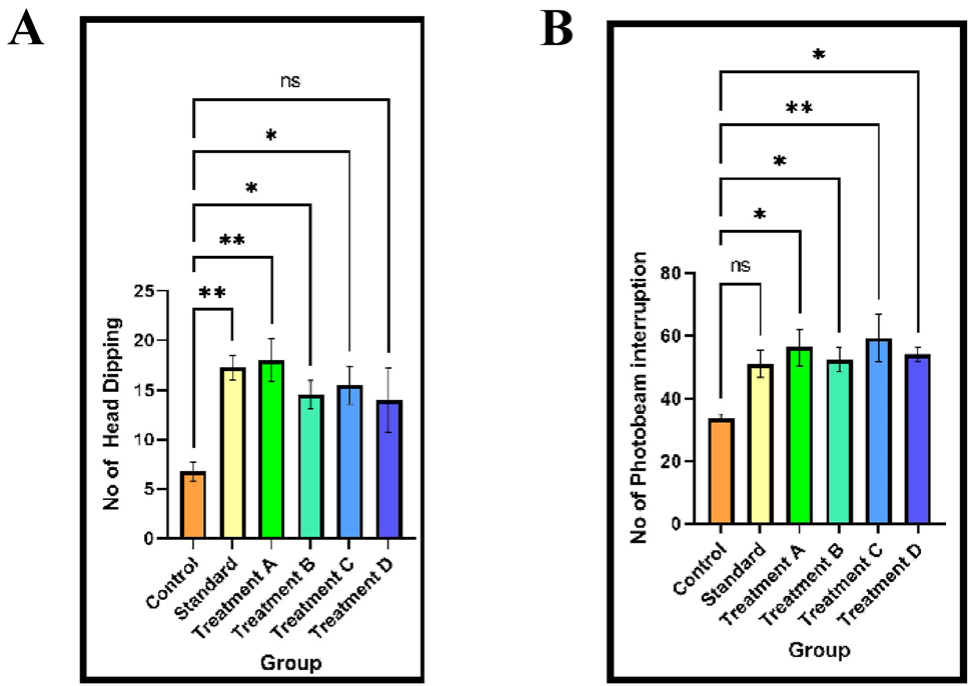
(**A**) The number of head dipping in a 5 min session of hole board was recorded. (**B**) The number of photo beam interruption in a 5 min session of actophotometer was recorded.

Figure 7A shows that the anxiolytic impact of the oral dose of 50 mg/kg led to a significant (p < 0.05) increase in the number of head dips. Moreover, the oral administration of 1 mg/kg clonazepam notably elevated the frequency of head dipping.

#### Locomotor activity (actophotometer)

The findings depicted in (Fig. 7B) exhibit the anxiolytic effects of flavan-3-ols and aromatic resin. The quantity of interruptions in the photo beams significantly increased (p < 0.05) upon oral administration at a dosage of 50 mg/kg. Furthermore, the oral administration of clonazepam at 1 mg/kg led to a notable increase in the number of interruptions in the light beams (p < 0.05).

## Discussion

Anxiety, a pervasive psychological phenomenon, has emerged as a focal point of extensive investigation within the domain of psychiatric research, primarily owing to its global prevalence, affecting approximately one-eighth of the world's populace^41,42^. The etiology of anxiety disorders is intricately intertwined with the intricate interplay of the GABAergic and serotonergic systems. Furthermore, the regulatory mechanisms of the adrenergic and dopaminergic pathways have also been implicated in the complex etiology of anxiety-related conditions. Although benzodiazepine (BZA) compounds have long served as a cornerstone in the therapeutic arsenal against various manifestations of anxiety for more than four decades, their clinical use has been marred by the emergence of unfavorable adverse reactions. COMT (Catechol-*O*-methyltransferase) is involved in the breakdown of catecholamines, including dopamine, which plays a role in mood regulation. Variations in the COMT gene can influence enzyme activity, leading to differences in dopamine levels. Dopamine is involved in modulating the activity of GABAergic neurons, which are responsible for inhibitory neurotransmission in the brain. Dysfunction in the dopaminergic-GABAergic system has been implicated in various psychiatric disorders, including anxiety. SLC6A4 (Solute Carrier Family 6 Member 4) encodes the serotonin transporter protein, which regulates serotonin levels by reuptaking serotonin from the synaptic cleft. Serotonin is a neurotransmitter involved in mood regulation, and abnormalities in its function have been linked to anxiety disorders. SSRIs, such as fluoxetine and sertraline, work by blocking the reuptake of serotonin, thereby increasing its availability in the synaptic cleft. Genetic variations in SLC6A4 have been associated with altered responses to SSRIs and susceptibility to anxiety disorders. MAO-A (Monoamine Oxidase A) is an enzyme involved in the breakdown of neurotransmitters such as serotonin, dopamine, and norepinephrine. Variations in the MAO-A gene can influence enzyme activity, leading to differences in the metabolism of these neurotransmitters. Dysregulation of serotonin metabolism has been implicated in anxiety disorders, and MAO-A inhibitors are sometimes used as antidepressant medications. Genetic variations in MAO-A have been studied about anxiety susceptibility and response to treatment. The interactions between these genes and neurotransmitter systems are complex and multifaceted. For example, variations in COMT, SLC6A4, and MAO-A genes can influence the balance of neurotransmitters like dopamine and serotonin, which in turn can affect the activity of GABAergic neurons. Additionally, these genetic variations may impact individual responses to medications targeting these neurotransmitter systems, such as SSRIs.

As a result, alternative treatment modalities boasting favorable side effect profiles have garnered increased amounts of attention and implementation. Anxiety network delineation was facilitated by the identification and discernment of plant bioactive targets, followed by the discernment of targets linked with the anxiety pathway. The resultant network demonstrated the potential of bioactive substances to modulate anxiety through intricate interactions involving 526 proteins across multiple pathways. Using protein‒protein interaction (PPI) analysis, we identified 98 nodes and 95 edges. Notably, MAOA, MAOB, COMT, DRD2, HTR1A, ACHE, GPR55, and SLC6A4 were identified as pivotal genes within the context of anxiety regulation. Further investigation through Gene Ontology (GO) and Kyoto Encyclopedia of Genes and Genomes (KEGG) analyses revealed numerous pathways, in addition to highlighting associations with various other diseases and disorders involving the aforementioned genes. The GO enrichment analysis substantiated the direct implication of biological activity in the regulatory mechanisms underlying anxiety.

Furthermore, the KEGG pathway analysis underscores the critical role played by the serotonergic and dopaminergic signaling pathways within the designated network. This observation lends support to the proposition that flavan-3-ols and aromatic resin may serve as viable candidates for the treatment of anxiety. In addition to the known serotonergic and dopaminergic signaling pathways, our investigation revealed the involvement of an array of intricate molecular networks, including but not limited to, the resistance mechanism associated with the epidermal growth factor receptor (EGFR) tyrosine kinase inhibitor and the pathways implicated in neurodegeneration.

These findings collectively suggest a promising, multifaceted approach leveraging flavan-3-ols and aromatic resin as potential multitarget therapeutic agents. The results of the molecular docking analysis revealed an estimated range of free energies of binding for the docked ligands, varying from −6 to −9.2 kcal/mol for the serotonin transporter (SLC6A4), −6 to −9.5 kcal/mol for monoamine oxidase-A (MAO-A), and −5.8 to −7.2 kcal/mol for catechol-O-methyltransferase (COMT). The observed binding patterns between the bioactive compounds and the target proteins were attributed to a complex interplay of hydrophobic interactions, the formation of hydrogen bonds, and other critical interactions, including π-stacking and the creation of salt bridges. Interestingly, our data highlighted that the flavan-3-ol compounds, specifically epigallocatechin gallate, demonstrated the most pronounced affinity for all the aforementioned receptors within the experimental series. The superior binding affinity of epigallocatechin gallate can be attributed to its extensive interaction with a greater number of key amino acid residues. Notably, it was established that an increased number of hydrogen bonds corresponded to heightened inhibitory potential. Further meticulous examination of the docking scores substantiated that epigallocatechin gallate exhibited a compelling binding energy of −9.2 kcal/mol toward the serotonin transporter in stark comparison to the standard clonazepam, which displayed a binding energy of −6.9 kcal/mol.

Epigallocatechin gallate was found to engage in the formation of 9 significant hydrogen bonds with crucial amino acid residues, such as SER224, ARG390, GLU392, ASP400, SER404, THR409, ARG564, LEU565, and GLN567, in contrast to the standard compound. Similarly, the binding energy of epigallocatechin gallate toward MAO-A was determined to be −9.5 kcal/mol, in stark contrast to the reference clonazepam, which has a binding energy of −6 kcal/mol. This interaction was facilitated by the formation of 7 pivotal hydrogen bonds with essential interacting amino acid residues, namely, ASN179, GLU185, ASP328, GLU329, SER334, LEU354, and LYS357. Similarly, the binding energy of epigallocatechin gallate toward COMT was recorded as −7.2 kcal/mol in comparison to that of the standard clonazepam, which has a binding energy of −5.8 kcal/mol. This interaction involved the formation of 4 significant hydrogen bonds with critical interacting amino acid residues, namely, LYS55, ASP191, LYS194, and ASN220.

**Figure 8:**
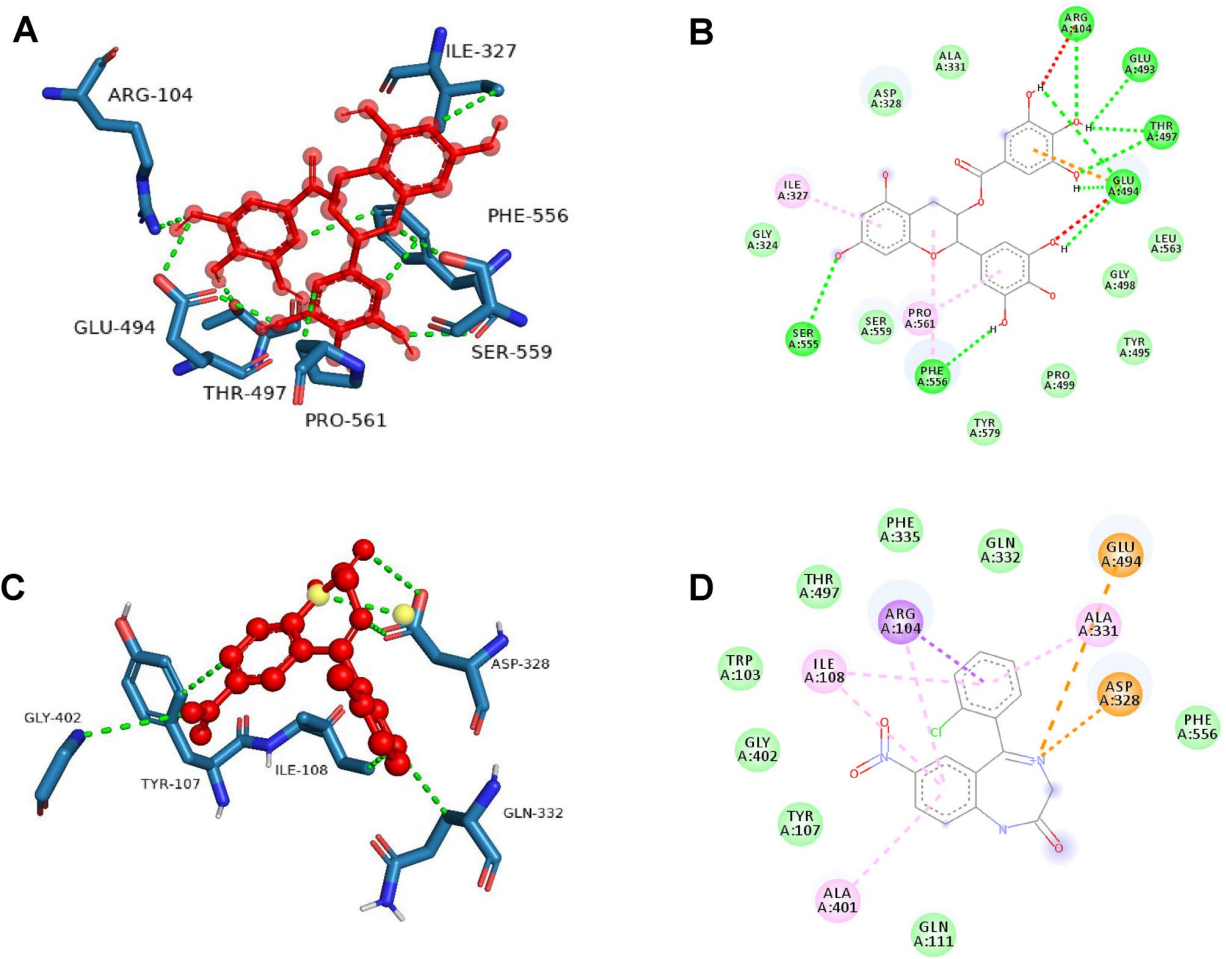
3D and 2D Amino acid interactions formed between docked complexes of EGCG and Clonazepam. (**A,B**) 3D and 2D interaction diagram of EGCG with 7lia. (**C,D**) 3D and 2D interaction diagram of standard clonazepam with 7lia.hydrogen bonds are represented in green dashed line where EGCG and standard clonazepam is presented in ball and stick.

The formation of hydrogen bonds and hydrophobic interactions plays a crucial role in determining the specificity of drug candidates with the active site residues of the target protein. Hydrogen bonds facilitate specific interactions between the ligand and the amino acid residues within the active site, contributing to the binding affinity and stability of the ligand–protein complex. Additionally, hydrophobic interactions between nonpolar regions of the ligand and the protein further enhance the binding specificity by optimizing the complementarity between the ligand and the active site. Overall, understanding and optimizing these interactions are essential for designing potent and selective drug candidates in molecular docking studies^43^. The amino acid interactions formed within docked complexes of 7LIA protein with EGCG (Fig. 8A, B) and standard drug Clonazepam (Fig. 8C, D) were analyzed and it is apparent from interaction analysis that TRP220, SER226, GLU229, ARG390, GLU392, LYS399 and ASP400 are forming hydrogen bonds with EGCG and GLU494 for standard clonazepam. The Amino acid residues ARG104 form a conventional H-bond bond with EGCG by donating an electron pair to the O3 atom of EGCG with a bond length of 2.21 Å. GLU494 forms 2 conventional H-bond bonds with O3 atom of EGCG with a bond length of 1.87 Å and 2.28 Å, and 1 conventional H-bond bonds with O– with a bond length of 2.69Å.While THR497, forms 2 conventional H-bond bonds with O3 atom of EGCG with a bond length of 2.64 Å and 2.15 Å. The amino acids PHE556 and SER559 each form 1 conventional H-bond bonds with O2 and O3 atoms with a bond length of 2.52 Å and 2.81 Å respectively in comparison with Clonazepam with less hydrogen bonding with amino acids. The EGCG forms 7 conventional H-bond bonds with 7LIA, suggesting that these hydrogen bonds are critical for stabilizing EGCG in the protein pocket. The ligand interaction fraction of clonazepam shows only one H-bond with GLU494 which indicates less stability in the binding pocket of the protein. Additionally, EGCG exhibited strong hydrophobic interactions with TRP220 and ILE408, which may also stabilize the complex, while standard clonazepam shows hydrophobic interactions with TYR107, ILE108, ALA331, ALA401, LYS419, and PHE556.

The chosen ligand exhibited the least binding energy, with critical binding residues identified as TRP220, THR225, SER226, GLU229, GLU392, LYS399, ASP400, and ILE408 observed with SLC6A4. The calculation of the root mean square deviation (RMSD) and root mean square fluctuation (RMSF) values provided insights into the deviation of the molecule from the active site. The lower RMSD and RMSF values obtained suggested that the drug-ligand complexes were stable and that the molecules did not exhibit significant fluctuations from the active site of the protein throughout the entire 100 ns MD simulation. The stability of the interactions in the docked complex was evident, as minimal fluctuations were observed during the entire 100 ns duration of the MD simulations. MM-GBSA analysis revealed increased binding of the ligands to the receptors. The outcomes of the investigation demonstrated that the constituent flavan-3-ols (epigallocatechin gallate) exhibited the most pronounced inclination toward all the examined receptors within the series. It is important to note that a greater number of hydrogen bonds corresponding translates to an augmented inhibitory potential. The determination of dynamic and thermodynamic parameters within living systems under specific physiological conditions can be ascertained through the utilization of MD simulations, a prevalent technique in computer-aided drug design^44^. To assess the stability of the drug-ligand docked complex, the top molecules were subjected to molecular dynamics simulation studies. The accuracy of the simulated complexes was consistent, particularly at the binding site of the target protein. Molecular docking analyses, in conjunction with MD simulation analyses, elucidated the interacting residues of the selected ligands and the receptor protein.

Our investigation revealed that the interaction between flavan-3-ols and aromatic resin compounds targets multiple neuroradios, notably, catecholamine *O*-methyltransferase (COMT), sodium-dependent serotonin transporter, and monoamine oxidase (MAOA). This complex interplay potentially underlies the anxiolytic properties of flavan-3-ols and aromatic resin, presumably accomplished through the modulation of the activities of these receptors. The comprehensive action of flavan-3-ols and aromatic resin compounds manifests through their selective influence on pivotal proteins involved in the neuroactive ligand-receptor interaction network, encompassing G protein-coupled receptors (GPCRs), MAOAs, COMTs, and serotonin. This observation highlights the potential of flavan-3-ols and aromatic resins as agents with pronounced anxiolytic efficacy. These insights substantiate the hypothesis that the anxiolytic effects of flavan-3-ols and aromatic resin arise from their regulation of diverse signaling pathways. Network analysis of protein–protein interactions revealed MAOA, COMT, and serotonin as critical components, further underscoring their significant contributions to the anxiolytic activity of flavan-3-ols and aromatic resin. The intricate functionality of various G protein-coupled receptors (GPCRs), activated by neurotransmitters such as serotonin, dopamine, and gamma-aminobutyric acid (GABA), assumes a pivotal role in the intricate web of signal transduction. Disruptions in these GPCR-mediated signaling cascades are strongly implicated in the etiology of psychiatric disorders, including anxiety and depression, as outlined in previous research^45^.

Neurotransmitter receptors, pivotal entities implicated in the pathophysiological mechanisms underlying psychiatric conditions such as anxiety and depression, primarily operate within the intricate network of neuroactive ligand-receptor interaction signaling pathways^46^. Through comprehensive analyses encompassing the construction of network pathways, as well as Gene Ontology (GO) enrichment analysis and Kyoto Encyclopedia of Genes and Genomes (KEGG) pathway enrichment analysis, our investigation revealed that the dynamic components of flavan-3-ols and aromatic resin hold potential therapeutic significance in the amelioration of anxiety. The literature emphasizes the fundamental role of biogenic amines within the receptor structures of the neuroactive ligand–receptor interaction signaling pathway.

These biogenic amines serve as crucial neurostimulatory molecules that orchestrate and modulate vital biological functions upon binding to their corresponding receptors. Notably, disturbances or downregulation of these receptors within the pathway culminate in the manifestation of anxiety-related symptoms^47,48^. Furthermore, our findings underscore the importance of the serotonin synaptic pathway, where serotonin, a well-known neurotransmitter renowned for its role in promoting a sense of well-being, is implicated in the regulation of mood, energy, and memory. The intricate interplay of serotonin is intimately associated with the etiology of anxiety disorders^49^.

Evidential support has also suggested that perturbations within the serotonergic system, characterized by aberrations in serotonin levels or disruptions in serotonin receptor activity, might contribute significantly to the onset and perpetuation of anxiety disorders. This assertion reinforces the observation of increased anxiety-related behaviors in animal models exhibiting diminished serotonin levels or impaired serotonin receptor function. Further elucidating the intricate interplay between neurotransmission and immune activation, alterations in serotonin 5-hydroxytryptamine (5-HT) signaling, in conjunction with peripheral immune activation, have been strongly associated with a spectrum of psychiatric disorders, revealing the complex and multifaceted nature of these underlying pathophysiological processes. Notably, SLC6A4 has emerged as a pivotal gene central to serotonin transport, and emerging research has highlighted the impact of various predisposing factors, including epigenetic mechanisms such as SLC6A4 promoter methylation and microRNA-mediated silencing, on affective disorders.

Notably, the potential anxiolytic effects of flavan-3-ols and aromatic resin may be mediated through the modulation of dopamine, serotonin, and norepinephrine pathways, further emphasizing the intricate interconnections between neurotransmitter systems and psychiatric well-being. Moreover, the intricate neuroactive ligand‒receptor interaction signaling pathways, closely intertwined with learning, memory, and various neuronal processes, such as neural plasticity and synaptic function, underscore the intricate network of molecular players contributing to the dynamic neural landscape. Concomitantly, investigations into the potential antianxiety activities of flavan-3-ols and aromatic resin were conducted via the oral administration of these compounds in a murine model, followed by a comprehensive evaluation of behavioral outcomes. Notably, emerging evidence has demonstrated the favorable impact of flavan-3-ols and aromatic resin on anxiety-related behaviors, further underscoring the potential therapeutic utility of these compounds within the domain of mental health management.

In the realm of experimental behavioral analysis, a series of well-established methodologies were employed, namely, the elevated plus-maze (EPM) test, light and dark test, open field test, hole board test, and locomotor activity analysis via an actophotometer. Within the context of the EPM test, a comparative investigation of the anxiolytic properties of flavan-3-ols and an aromatic resin was performed. Compared with those in the control group, the 50 mg/kg dose substantially increased the number of entries into the open arms and the duration of time spent in the open arms (p < 0.05). Conversely, a notable reduction was observed in both the number of entries into closed arms and the duration of time spent within those areas (p < 0.05). Remarkably, the application of clonazepam at a dosage of 1 mg/kg induced a considerable surge in the number of entries and the time spent in the open arms, while simultaneously eliciting a significant decrease in the number of entries and the time spent in the closed arms (p < 0.05). Consequently, ingrowth into the open arms can be interpreted as indicative of a holistic invigorating, and anxiety-reducing behavioral pattern.

Moreover, through the implementation of a light and dark test, the present investigation further elucidated the anxiolytic potential of flavan-3-ols, aromatic resins, and clonazepam in murine models. The light and dark test represents an established technique for the assessment of anxiolytic drug efficacy^36^. Notably, at a dosage of 50 mg/kg, there was a significant (p < 0.05) increase in the duration spent within the light compartment. Similarly, clonazepam at 1 mg/kg also elicited a considerable (p < 0.01) escalation in the time spent within the light compartment. Additionally, a conspicuous reduction in both the temporal extent of residency and the frequency of entries into the shaded compartment was discerned at 50 mg/kg, thereby implying substantial mitigation of the anxiety levels of the experimental subjects after the designated treatments. Consequently, the combined administration of flavan-3-ols and aromatic resin demonstrated a notable anxiolytic effect according to the results of the light and dark box tests.

The open field test was conducted to elucidate the emotional state of the subject animal. This experimental framework serves to probe anxiety-associated behavioral patterns delineated by the innate reluctance of the animal to confront an exposed milieu. Consequently, upon being translocated from their accustomed enclosure to an unaccustomed setting, subjects manifest indications of perturbation and apprehension through modulations in one or more measurable parameters. Remarkably, murine cohorts subjected to the composite treatment regimen exhibited a discernible escalation in the temporal extent of central zone occupancy and the frequency of entries therein. Concomitantly, a noticeable reduction in both the duration spent within the peripheral zones and the frequency of entries into these areas was observed.

Each specimen within the murine cohort was individually introduced into the orifice panel, and the number of cranial protrusions was meticulously documented. The oral administration of a confluence of flavan-3-ols and aromatic resin at a dosage of 50 mg per kilogram was observed to elicit a substantial escalation in the tally of cephalic immersions, displaying statistical significance at a significance level of p < 0.05. Correspondingly, the oral administration of clonazepam at a dosage of 1 mg/kg, serving as the benchmark pharmaceutical agent, also exhibited a notable augmentation in exploratory conduct. These results suggest the potential anxiolytic efficacy of the aforementioned combinations. The monitoring of locomotive activity is acknowledged as a key determinant of vigilance, with a reduction in locomotion denoting a sedative influence. The aforementioned combination was effective at enhancing the perceptiveness of the animals, thus underscoring the anxiolytic attributes of the compound amalgamation. Some recent literature suggested that there is a potential correlation between anxiety and neurodegenerative diseases like Alzheimer's disease (AD) and Parkinson's disease (PD). AD and PD patients often experience higher levels of anxiety, cognitive decline, and impaired motor symptoms compared to the general population which may lead to disease progression. The present results of Flavan-3-ols and aromatic resin may contribute to preventing the neurodegenerative disease progression through anxiolytic action. Early identification and targeted management of anxiety symptoms concerning AD and PD may contribute to better overall patient care and quality of life. These comprehensive findings establish a foundation for further investigations and the potential clinical application of flavan-3-ols and aromatic resins in anxiety treatment.

## Conclusion

In the present investigation, we applied a comprehensive approach, encompassing network pharmacology integrated with extensive database mining techniques, to elucidate the principal target proteins associated with flavan-3-ols and aromatic resins for the amelioration of anxiety disorders. This involved the establishment of a meticulously constructed network of target proteins. Notably, our findings revealed that the engagement of flavan-3-ols and aromatic resin primarily occurs within the domains of neuroactive ligand‒receptor interactions, serotonin synapses, dopaminergic pathways, and other pertinent biological pathways. These actions were shown to modulate crucial target proteins, including SLC6A4, COMT, and MAOA, thereby exhibiting a significant therapeutic impact on anxiety disorders. Furthermore, we explored the intricate mechanism through which flavan-3-ols and aromatic resins effectively regulate anxiety by targeting multiple proteins and exerting influence through diverse signaling pathways. Behavioral tests demonstrated significant reductions in anxiety-like behavior, as indicated by increased time in the open arm (p < 0.05, EPM test), time in the light region (p < 0.01, light–dark test), and number of entries in the central area (p < 0.01, open field test). The hole board test and actophotometer recorded substantial increases in head dips (p < 0.01) and photobeam interruptions (p < 0.05), respectively. Together, the results of network analysis and behavioral studies strongly support the anxiolytic effects of these compounds, indicating their potential as novel pharmacological interventions in anxiety management. Further research is essential to deepen our understanding and develop effective anxiolytic strategies.

### Future perspective

The study presented a comprehensive approach to investigating the anxiolytic properties of flavan-3-ols and aromatic resins. To validate and apply these findings, future research should focus on in vitro studies. Further advancements in network pharmacology, molecular docking, and experimental verification techniques. This could lead to a deeper understanding of how flavan-3-ols and aromatic resin affect anxiety at a molecular level. Clinical trials are also necessary to assess their safety and effectiveness in treating anxiety disorders in humans.

### Supplementary Information

Supplementary Figures.

Supplementary Tables.

Supplementary Table S3.

## Data Availability

The datasets used and/or analyzed during this study are available from the corresponding author on reasonable request.

## Abbreviations

KEGG: Kyoto Encyclopedia of Genes and Genomic analysis
GABA: Gamma-aminobutyric acid
COMT: Catechol-*O*-methyltransferase
LD50: Lethal dose 50
P.O: Per oral/orally
OECD: Organization for Economic Cooperation and Development
PPI: Protein–protein interaction
PDB: Protein Data Bank
EPM: Elevated plus maze
h: Hour
ANOVA: Analysis of variance
MAOA: Monoamine oxidase A
HTR1A: 5-Hydroxytryptamine receptor 1A
HTR6: 5-Hydroxytryptamine receptor 6
GPR55: G protein-coupled receptor 55
Kcal/mol: Kilocalories per mole
BZA: Benzodiazepines
EGCG: Epigallocatechin gallate
GPC: G protein-coupled receptors
DR: Dopamine receptor
HTR: 5-HT receptor
SLC6A4: Solute Carrier Family 6 Member 4
°C: Degree Celsius
L/D: Light and dark
MD: Molecular dynamic
