# Supplementary material for: Network pharmacology combined with molecular docking and experimental verification to elucidate the effect of flavan-3-ols and aromatic resin on anxiety
Source: Sci Rep. 2024 Apr 29;14:9799. doi: 10.1038/s41598-024-58877-z (PMC11058257; doi:10.1038/s41598-024-58877-z)
Supplement: Supplementary file 1 — Supplementary Figures. [file 41598_2024_58877_MOESM1_ESM.pptx]

## Slide 1
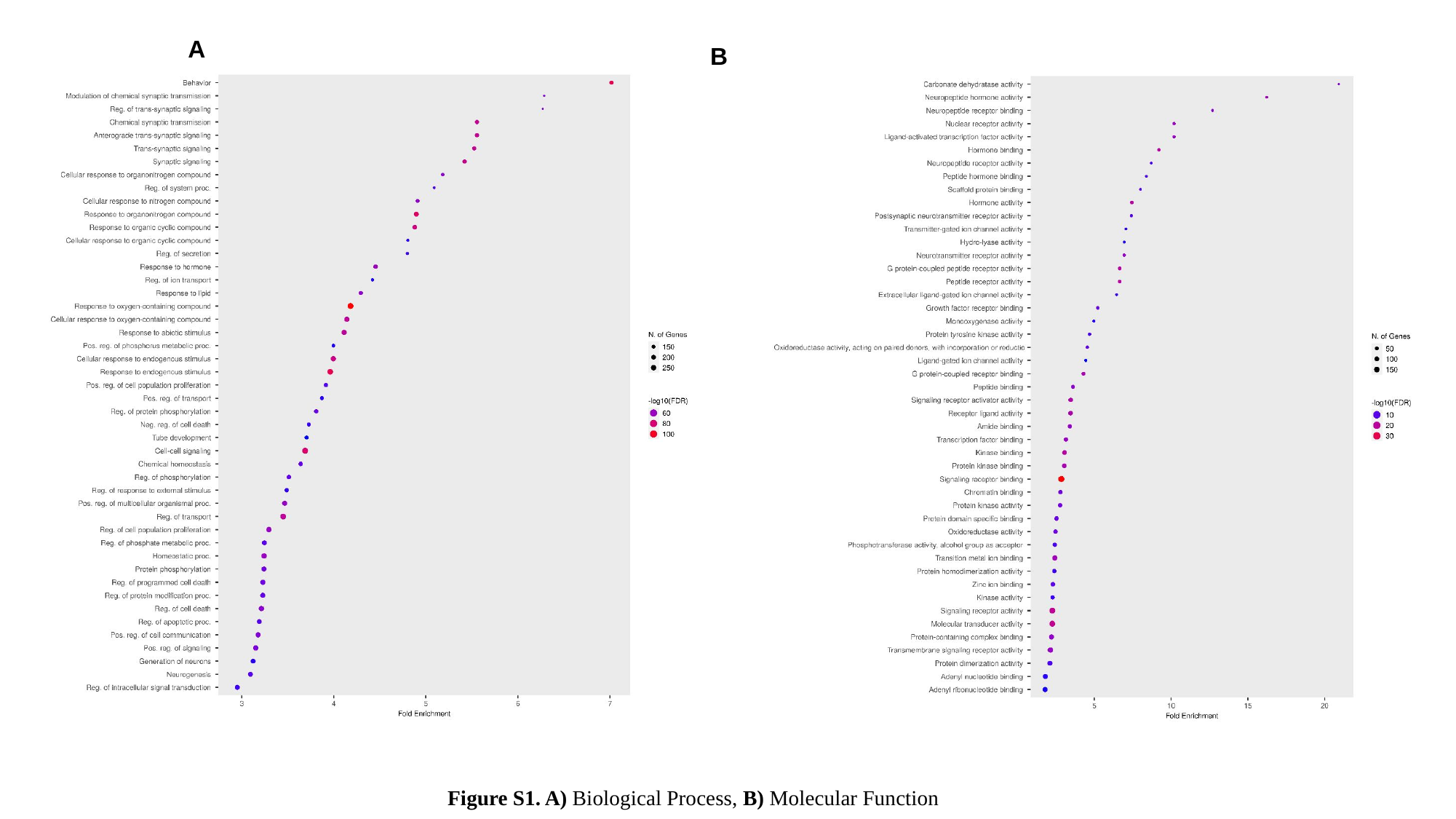

A
B
Figure S1. A) Biological Process, B) Molecular Function

## Slide 2
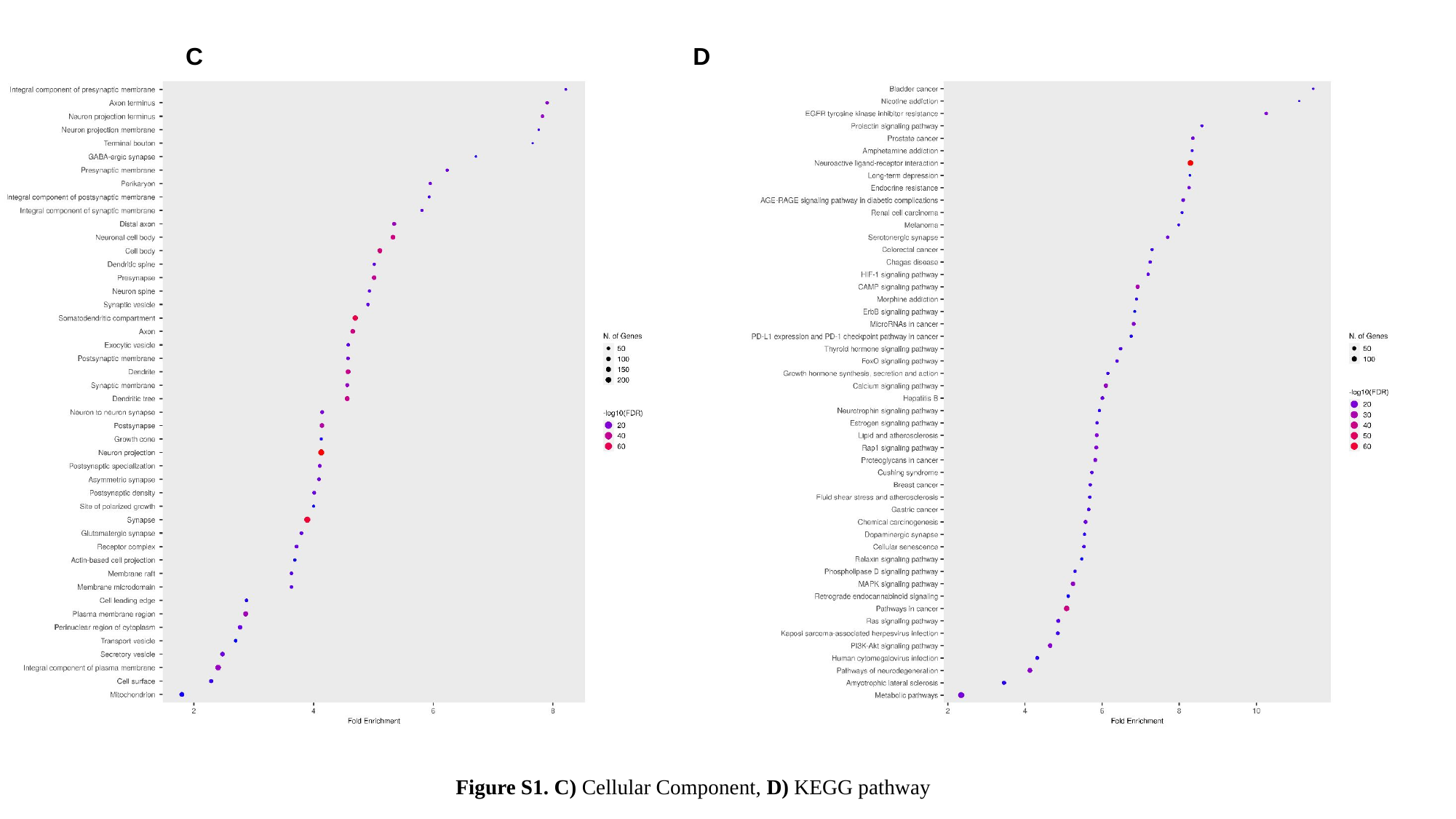

C
D
Figure S1. C) Cellular Component, D) KEGG pathway

## Slide 3
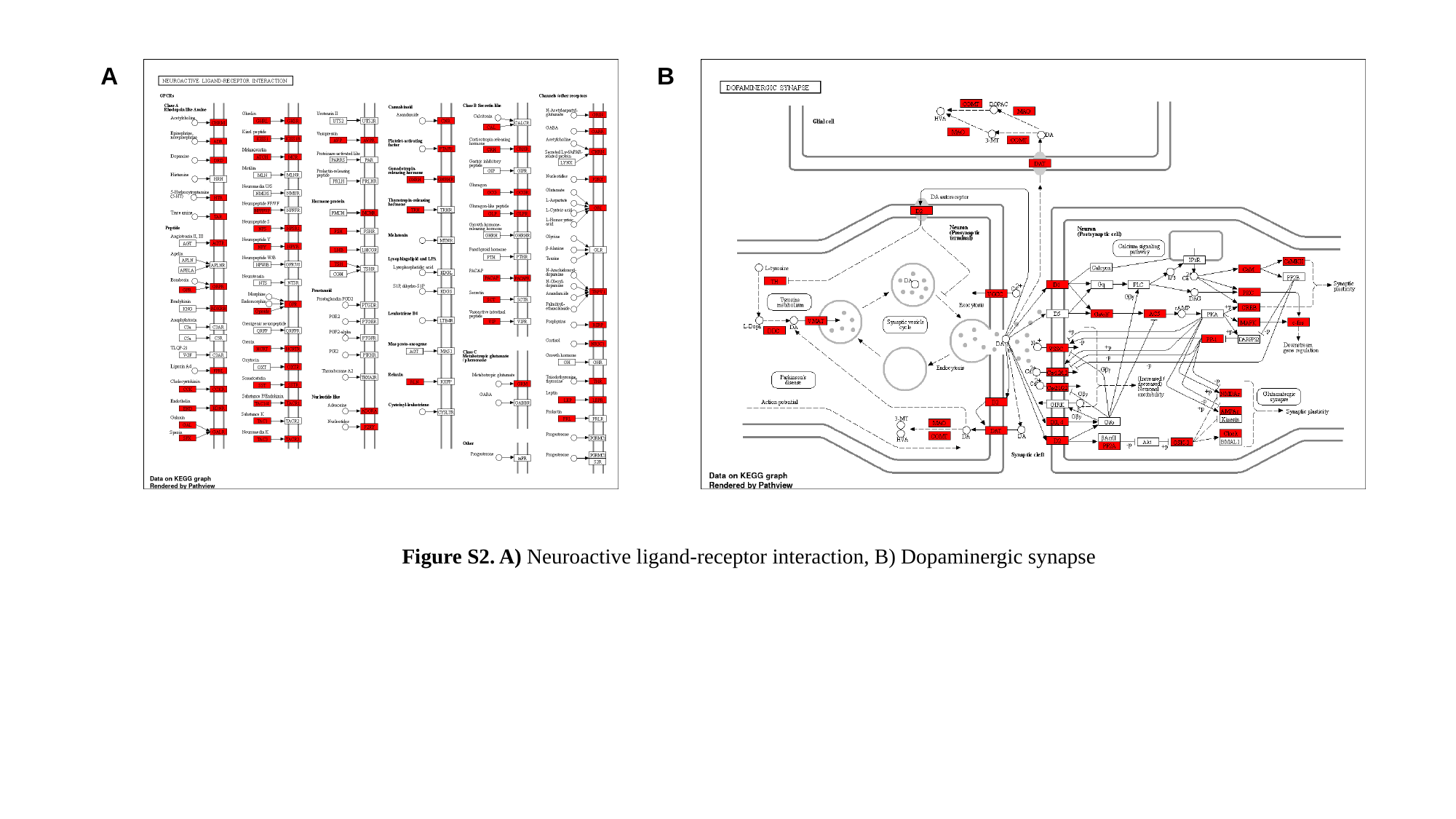

A
B
Figure S2. A) Neuroactive ligand-receptor interaction, B) Dopaminergic synapse

## Slide 4
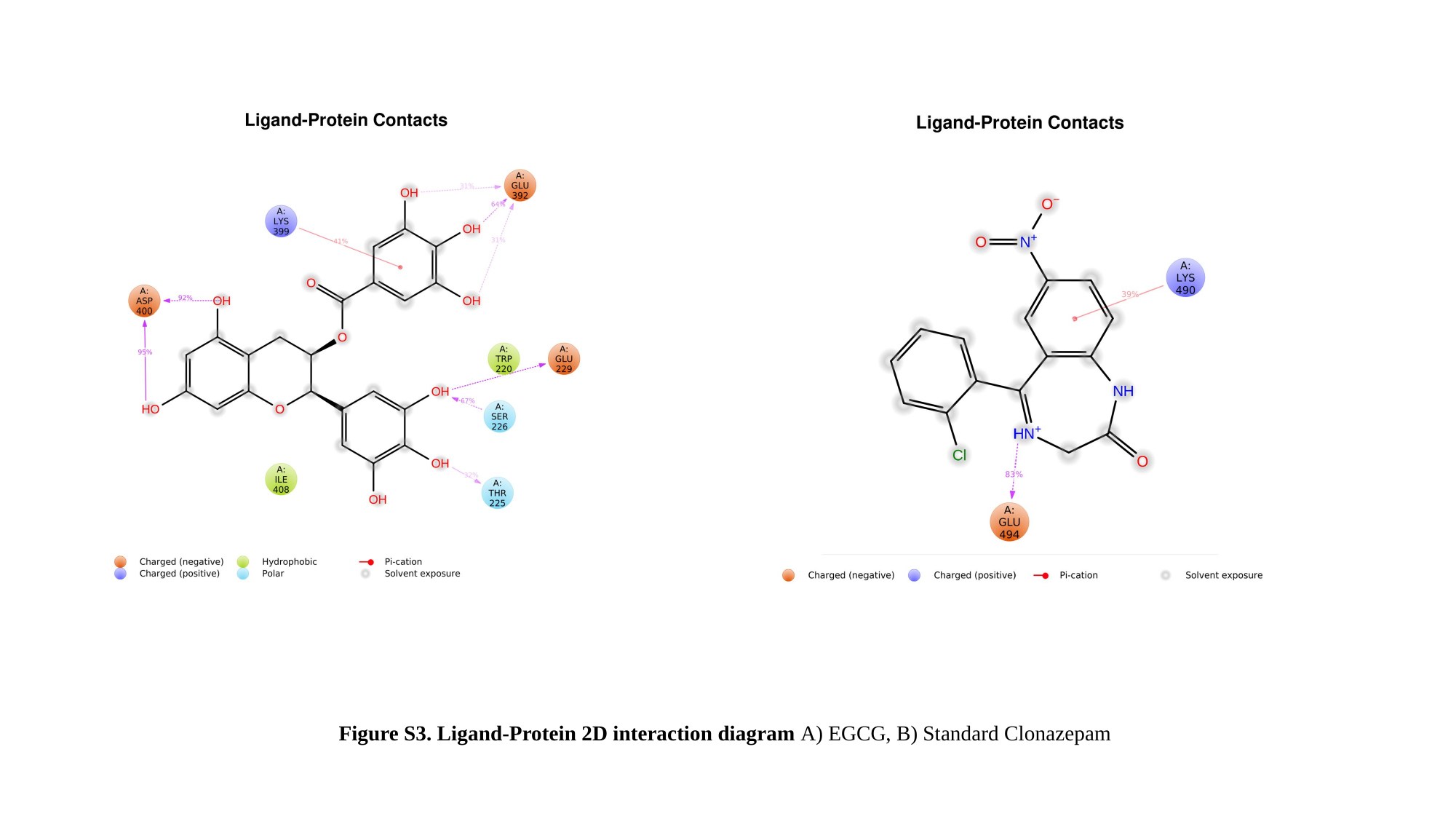

Figure S3. Ligand-Protein 2D interaction diagram A) EGCG, B) Standard Clonazepam

## Slide 5
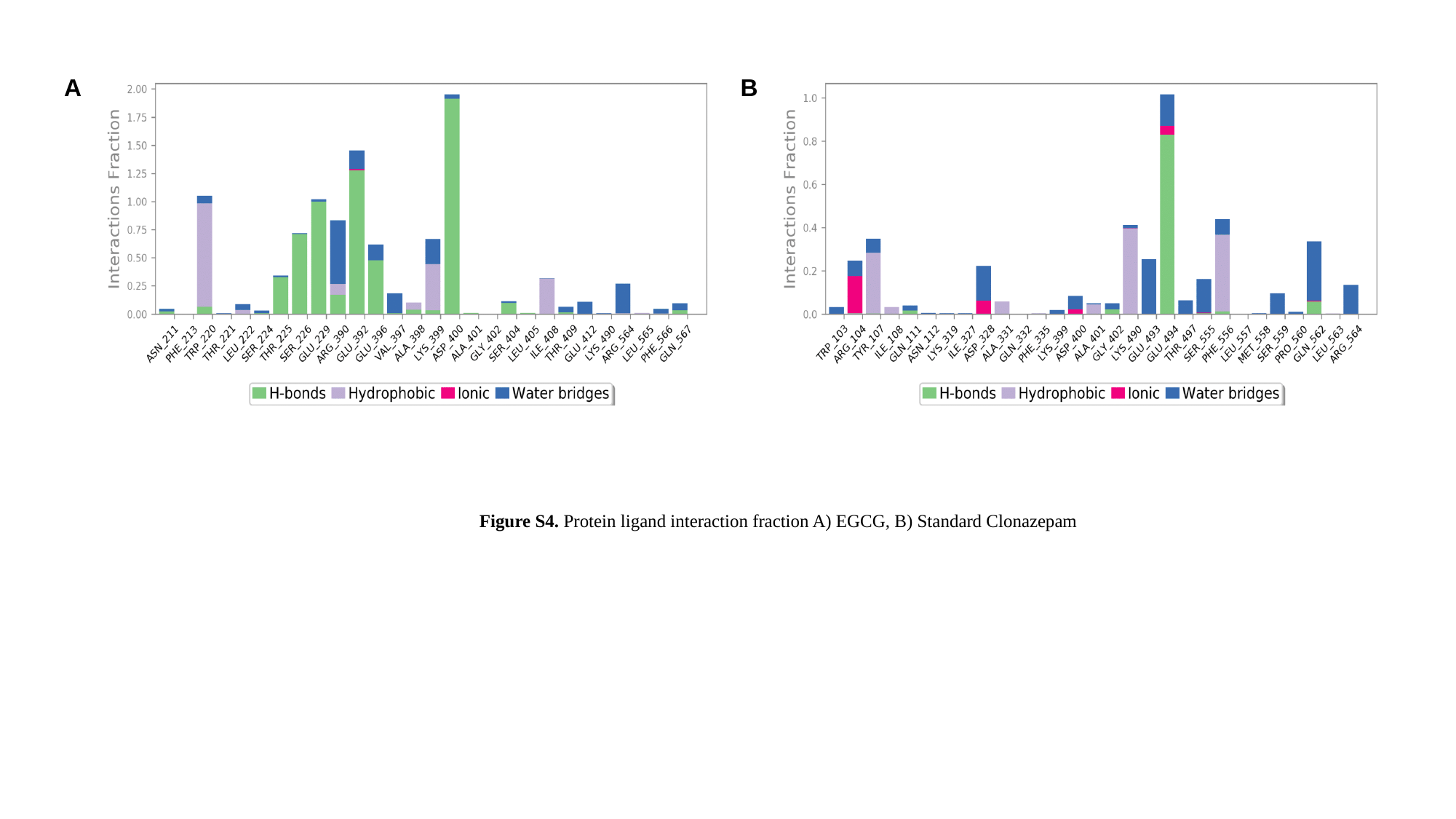

A
B
Figure S4. Protein ligand interaction fraction A) EGCG, B) Standard Clonazepam

## Slide 6
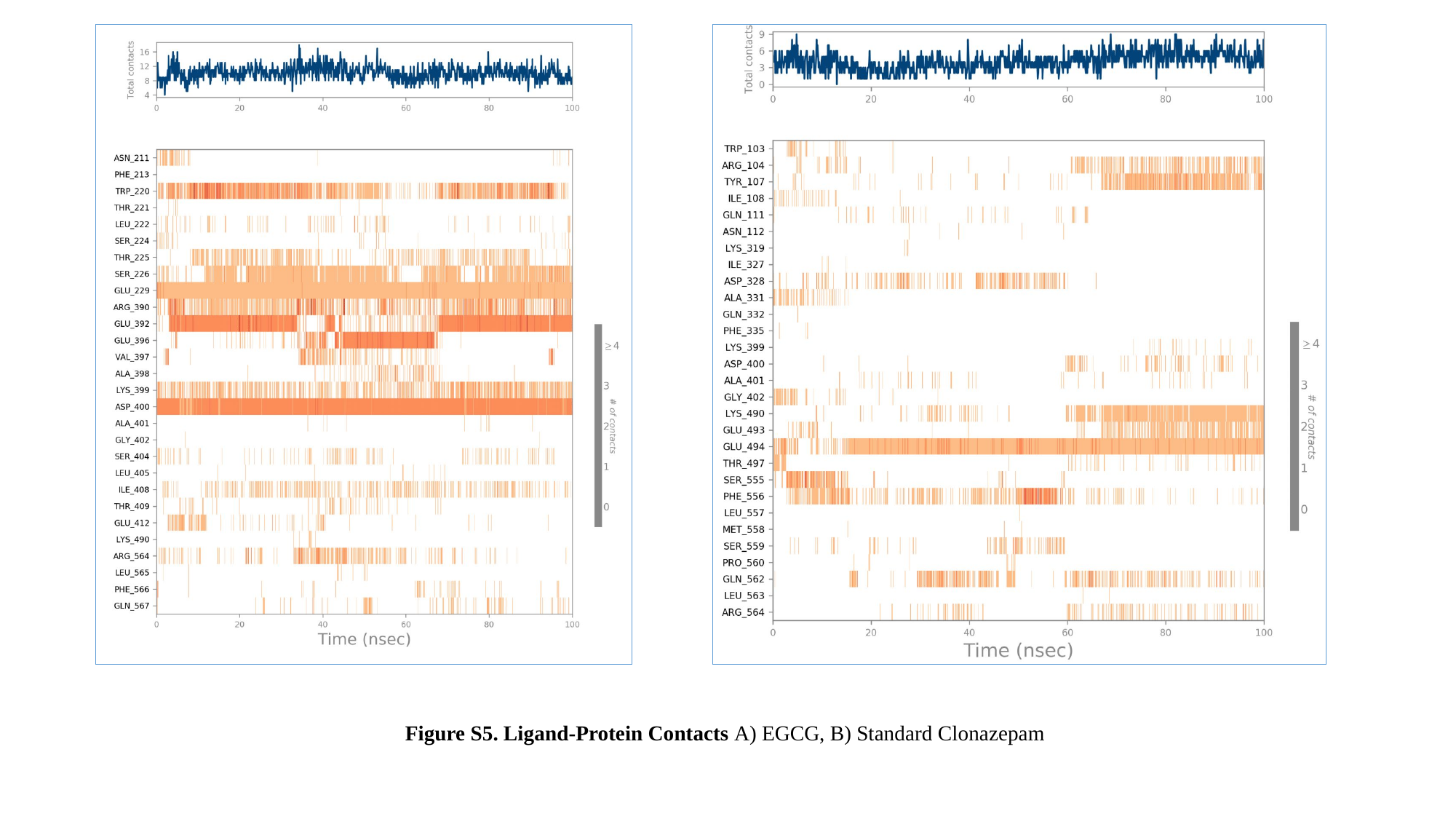

Figure S5. Ligand-Protein Contacts A) EGCG, B) Standard Clonazepam

## Slide 7
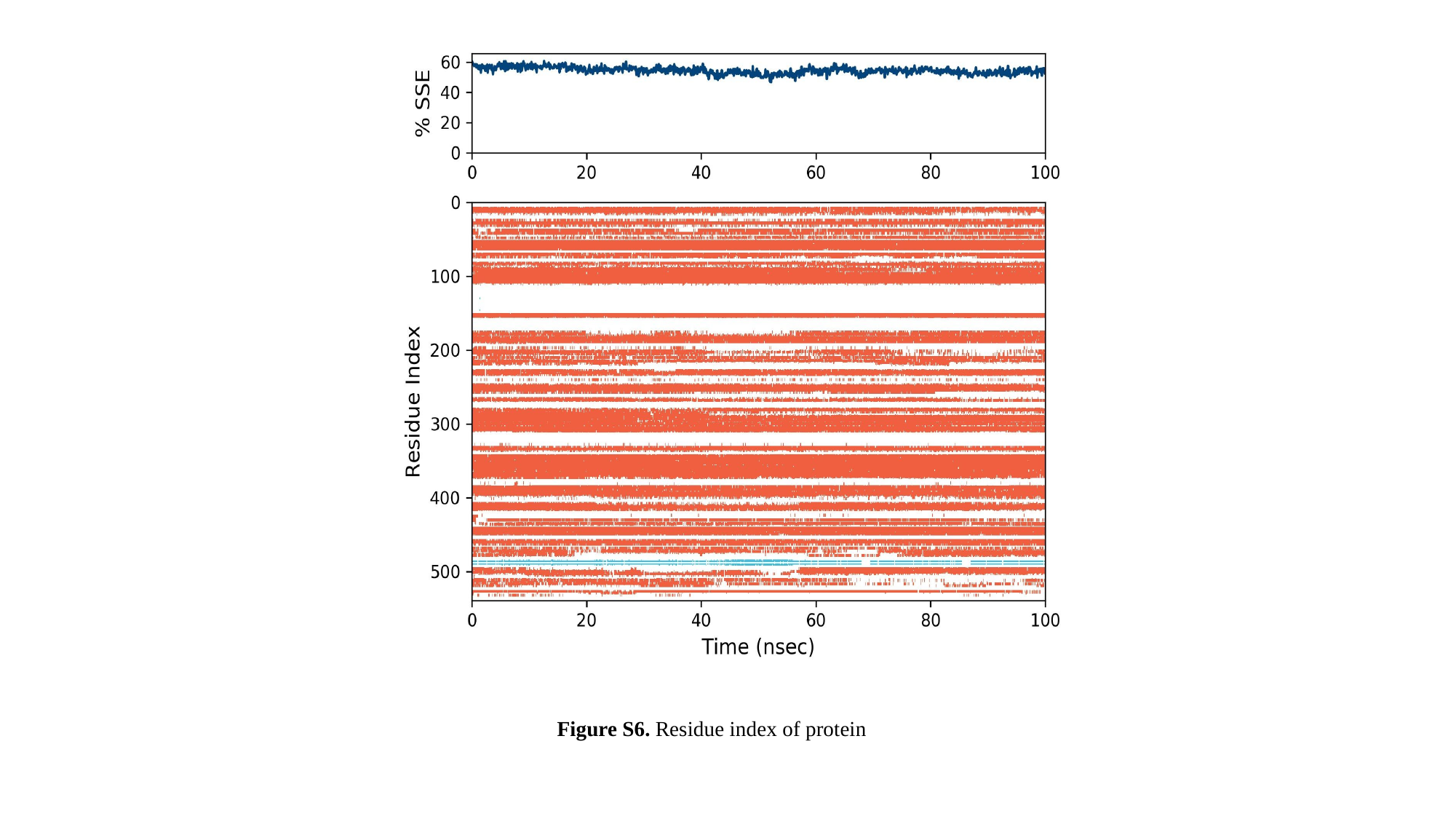

Figure S6. Residue index of protein

## Slide 8
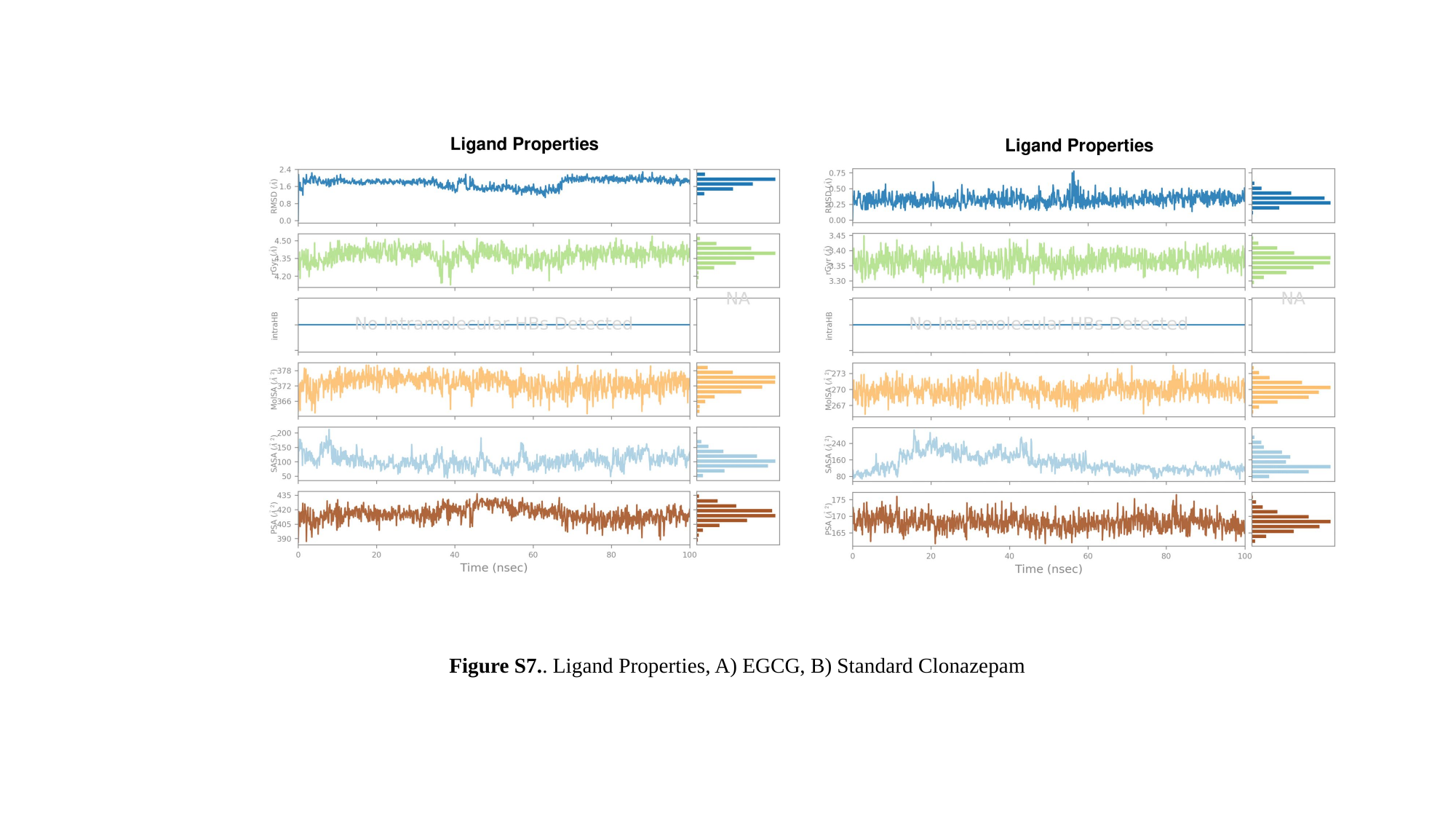

Figure S7.. Ligand Properties, A) EGCG, B) Standard Clonazepam
