# Supplementary material for: Network pharmacology combined with molecular docking and experimental verification to elucidate the effect of flavan-3-ols and aromatic resin on anxiety
Source: Sci Rep. 2024 Apr 29;14:9799. doi: 10.1038/s41598-024-58877-z (PMC11058257; doi:10.1038/s41598-024-58877-z)
Supplement: Supplementary file 2 — Supplementary Tables. [file 41598_2024_58877_MOESM2_ESM.docx]

**Table S1** Experimental Protocol

| **Group** | **Treatment** |
| --- | --- |
| A | Normal control group p.o. (Vehicle 0.1ml) |
| B | Standard (Clonazepam) p.o. (1 mg/kg) |
| C | Treatment A p.o. (Catechin + Epigallocatechin gallate + aromatic resin) |
| D | Treatment B p.o. (Catechin + Epigallocatechin gallate) |
| E | Treatment C p.o. (Plain aromatic resin) |
| F | Treatment D p.o. (Catechin + Epigallocatechin gallate + aromatic resin + Clonazepam) |

**Table S2** Acute toxicity study

| **Sr. No** | **Response** | **Head** | | **Body** | | **Tail** | |
| --- | --- | --- | --- | --- | --- | --- | --- |
|  |  | **Before** | **After** | **Before** | **After** | **Before** | **After** |
| 1 | Alertness | Normal | Normal | Normal | Normal | Normal | Normal |
| 2 | Grooming | Absent | Absent | Absent | Absent | Absent | Absent |
| 3 | Restlessness | Absent | Absent | Absent | Absent | Absent | Absent |
| 4 | Touch response | Absent | Absent | Absent | Absent | Absent | Absent |
| 5 | Touch response | Normal | Normal | Normal | Normal | Normal | Normal |
| 6 | Pain response | Present | Absent | Present | Absent | Present | Absent |
| 7 | Tremors | Absent | Absent | Absent | Absent | Absent | Absent |
| 8 | Convulsion | Absent | Absent | Absent | Absent | Absent | Absent |
| 9 | Righting reflux | Normal | Normal | Normal | Normal | Normal | Normal |
| 10 | Gripping strength | Normal | Normal | Normal | Normal | Normal | Normal |
| 11 | Pinna reflux | Present | Present | Present | Present | Present | Present |
| 12 | Corneal reflux | Present | Present | Present | Present | Present | Present |
| 13 | Writhing | Absent | Absent | Absent | Absent | Absent | Absent |
| 14 | Pupils | Normal | Normal | Normal | Normal | Normal | Normal |
| 15 | Urination | Normal | Normal | Normal | Normal | Normal | Normal |
| 16 | Salivation | Normal | Normal | Normal | Normal | Normal | Normal |
| 17 | Skin Color | Normal | Normal | Normal | Normal | Normal | Normal |
| 18 | Lacrimation | Normal | Normal | Normal | Normal | Normal | Normal |

**Table S4**

The relative binding-free energies (kcal/mol) obtained by MM–GBSA, where MMGBSA dG Bind = Complex – Receptor – Ligand and MMGBSA dG Bind (NS) = Complex – Receptor (from optimized complex) – Ligand (from optimized complex) = MMGBSA dG Bind − Receptor Strain − Ligand Strain. NS in the table is no strain; it is the binding energy without considering for the receptor and ligand conformational changes needed for the formation of complex.

| **Compound** | **Time (ns)** | **MMGBSA-dG-binding energy** | **MMGBSA-dG-bind in Coulomb** | **MMGBSA-dG-bind(NS)** | **MMGBSA-dG bind(NS)-Coulomb** |
| --- | --- | --- | --- | --- | --- |
| EGCG | 0 | -69.1005 | -44.9757 | -87.5461 | -44.7549 |
| EGCG | 100 | -54.9226 | -42.3561 | -65.0752 | -46.473 |
| STD (Clonazepam) | 0 | -50.8769 | 42.6303 | -51.6052 | 42.4050 |
| STD (Clonazepam) | 100 | -35.9081 | 28.5823 | -36.4898 | 28.7585 |
